# Sunn Pest Optimization (SPO): A Phase-Differentiated Bio-Inspired Metaheuristic for Global Optimization with Application to Precision Agriculture Monitoring Networks

**DOI:** 10.3390/biomimetics11090676

**Published:** 2026-09-19

**Authors:** Samet Diri

**Affiliations:** Department of Information Systems Engineering, Kocaeli University, Kocaeli 41001, Türkiye; samet.diri@kocaeli.edu.tr

**Keywords:** bio-inspired computation, swarm intelligence, stagnation management, population diversity, Lévy flight, monitoring station placement

## Abstract

Metaheuristic optimization increasingly requires search operators with functionally justified biological grounding rather than superficial metaphors. This paper introduces Sunn Pest Optimization (SPO), a novel algorithm derived from the bioecology of the cereal pest *Eurygaster integriceps*. SPO couples directional Lévy flight exploration with differential swarm exploitation while utilizing a tiered stagnation hierarchy and a spatial diversity-override mechanism to manage population density. The algorithm was evaluated against seven classical and modern baselines across 16 CEC 2017 and CEC 2022 benchmark functions. At ten dimensions, SPO achieved the second-best average rank behind the state-of-the-art L-SHADE, outperforming WOA, GWO, and GBFIO in corrected pairwise comparisons while showing a mixed record against GA, PSO, and DE. Scalability tests at thirty dimensions showed that SPO retains its edge over several swarm-based methods but loses parity with classical evolutionary approaches. To assess practical readiness, the algorithm was applied to a budget-limited precision agriculture task involving the spatial placement of pest scouting stations. Although SPO achieved lower numerical coverage than the top performers, it reliably satisfied logistical constraints and successfully concentrated monitoring resources in high-risk zones to support integrated pest management. SPO is a transparent, biologically coherent metaheuristic with clearly defined scalability boundaries and demonstrated applicability to constrained real-world spatial design.

## 1. Introduction

Most real-world problems in domains such as precision irrigation scheduling, autonomous agricultural vehicle routing, agrochemical dosage optimization, and crop yield prediction feature high-dimensional, multimodal, and non-differentiable search spaces. While classical gradient-based methods fail in these problems, nature-inspired metaheuristic algorithms offer a powerful alternative [1].

The metaheuristic domain has grown rapidly since 1975. While genetic algorithm [2], particle swarm optimization [3], and differential evolution [4] constitute the cornerstones of this field, more recent, distinctive biological metaphors such as Grey Wolf Optimizer [5], Whale Optimization Algorithm [6], and Cuckoo Search [7] have found extensive application. Nevertheless, Wolpert and Macready’s “No Free Lunch” [8] theorem demonstrates that no algorithm can achieve universal superiority across all problem classes, which renders the development of new algorithms a constantly relevant research topic.

In this study, a novel algorithm inspired by the bioecology of the Sunn pest (*Eurygaster integriceps*), one of the most significant pests of wheat agriculture in the Caucasus, Middle East, and Turkey, is proposed. The seasonal vertical migration of the Sunn pest between mountain overwintering sites and lowland fields, the individual selective pressure of the entomopathogenic fungus Beauveria bassiana, and egg destruction by Trissolcus species Parasitoids provide a rare biological framework for modeling the exploration–exploitation balance. The mathematical abstraction of these three behaviors forms the foundation of Sunn Pest Optimization (SPO). Unlike existing metaheuristic methods, the SPO offers three original contributions to the literature. These are:

1. Phase-differentiated hybrid update rule using differential swarm vector with Lévy flight based on seasonal migration phases;
2. Three-layered stagnation hierarchy reflecting the ecological pressure tiers of Carabid, Beauveria, and Parasitoid;
3. Diversity-override mechanism that directly uses the spatial diversity of the population as an operator trigger.

Elitism (the overwintering memory mechanism) is a necessary fourth functional component of SPO but is not counted among the three original structural contributions listed above, since low-ratio elitism is a well-established design element in the broader metaheuristic literature rather than a novel mechanism. This distinction between the algorithm’s four functional components and its three original contributions is maintained consistently throughout the paper and is restated in Section 5.

The remainder of this paper is organized as follows: Section 2 reviews the related work; Section 3 presents the mathematical model, pseudocode, and design rationales of the proposed method, with biological background; Section 4 includes the experimental results, discussion, and limitations of the proposed method; and Section 5 concludes the paper.

## 2. Literature Review

### 2.1. Basic Metaheuristic Algorithms and Their Motivations

Optimization problems in engineering, science, and industry are becoming increasingly complex, multimodal, and nonlinear in structure. The tendency of traditional mathematical and derivative-based methods to get stuck in local optima in such challenging problems and their frequent inadequacy have led researchers toward flexible, nature-inspired, and gradient-free metaheuristic algorithms.

In recent years, although hundreds of different metaheuristic algorithms have been proposed in the literature, and there has been a significant increase in this field, the “No Free Lunch” (NFL) theorem, introduced by Wolpert and Macready [8], remains the most fundamental mathematical basis guiding optimization research. This theorem mathematically proves that a single universal algorithm that always yields the best result for all conceivable optimization problems cannot exist. In other words, while an algorithm may perform extremely well for a certain class of problems, it can fail for another. The NFL theorem always legitimizes the development of new metaheuristic algorithms with different exploration and exploitation balances that may outperform the existing methods.

The expanding metaheuristic literature is generally classified into four main categories according to the natural mechanisms that inspire them: evolutionary-based, swarm intelligence-based, physics/mathematics-based, and human-based methods. While genetic algorithm [2] and differential evolution [4], which are the fundamental building blocks of evolutionary algorithms, employ the principles of natural selection, popular methods such as particle swarm optimization [3], Artificial Bee Colony [9], and Grey Wolf Optimization [5] represent swarm intelligence approaches that model the social and collective behaviors of living creatures.

### 2.2. Current Metaheuristic Approaches and the Exploration/Exploitation Balance

Comprehensive review studies conducted in recent years [10,11] highlight that metaheuristic research is gaining momentum but also emphasize the risk that many proposed next-generation algorithms may only be minor modifications of the original approaches (PSO, GA, DE, etc.). Therefore, for a newly proposed algorithm to make a genuine contribution to the literature, it must offer an original and effective mechanism for balancing the broad search of the solution space, that is, exploration, with intensive search in promising regions, that is, exploitation.

In recent years, powerful original algorithms inspired by various sources have been introduced to the literature in order to achieve this balance. For example, the Sine Cosine Algorithm (SCA) [12], a major contribution to physics- and mathematics-based algorithms, has provided high exploration capability by utilizing the oscillatory properties of the trigonometric sine and cosine functions to update solutions in the search space. Among swarm-based methods, the Salp Swarm Algorithm (SSA) [13] has attracted attention by modeling the chain movements of salps in oceans and achieving rapid convergence in multimodal problems through leader and follower structures. Similarly, the Teaching–Learning-Based Optimization (TLBO) [14] algorithm, inspired by human behavior, presents a parameter-less original structure by modeling a teacher’s effort to increase students’ knowledge levels and the interactions among students in a class. Other approaches, such as the African Vultures Optimization Algorithm (AVOA) [15], inspired by the competitive feeding behavior of scavenger birds in nature, and the Grizzly Bear Fat Increase Optimization (GBFIO) [16], which is based on bears’ fat storage strategies before hibernation, have also been successfully applied to challenging engineering and design problems.

This trend toward highly specific, single-species biological metaphors has continued to accelerate in the recent literature. Examples include the Red-Crowned Crane Optimization algorithm [17], which models the courtship and foraging behaviors of Grus japonensis for engineering design problems; the Dual-Mechanism Enhanced Secretary Bird Optimization Algorithm [18], which augments an existing raptor-inspired metaphor with additional search operators, and the Improved Superb Fairy-Wren Optimization Algorithm [19], which refines a bird-behavior-based metaheuristic for numerical and applied optimization tasks. Complementing these single-metaphor designs, ref. [20] proposed two bio-inspired particle swarm optimization variants grounded in eavesdropping and altruistic animal behaviors, illustrating that even well-established frameworks such as PSO continue to be extended through biologically motivated operator design. Collectively, these contributions confirm that the search for functionally justified, rather than purely metaphorical, biological grounding—the central motivation of the present study—remains an active and unresolved concern within the current metaheuristic literature.

Nevertheless, regardless of how advanced the algorithms are, standard random walk or Gaussian-based mutation mechanisms may be inadequate for escaping local optimum traps in high-dimensional complex problems. To overcome this bottleneck, researchers have turned to more advanced statistical distributions and chaos theory, which introduce dynamism into the search process. For example, in the Chaotic Lévy Flight Distribution (CLFD) algorithm proposed by Yıldız et al. [21], chaotic maps such as Logistic, Tent, and Sine have been integrated into the standard Lévy flight. The CLFD model produced much more robust results than the Gaussian distribution by preventing the algorithm from becoming trapped in local optima during the exploitation phase in complex mechanical design problems.

### 2.3. Lévy Flights and Their Integration into Algorithms

Lévy flights, a strong alternative to traditional randomization principles, are defined as non-Gaussian stochastic processes whose stationary increments are distributed according to the Lévy stable distribution, named after the French mathematician Paul Pierre Lévy [22]. The key feature of this distribution is its heavy-tailed structure: the tail approaches zero much more slowly than that of a Gaussian distribution, allowing the production of rare but extremely large jumps interspersed among many small steps [23]. This statistical structure provides a distinct advantage over standard Gaussian-based random walks in terms of the ability to escape local optima. It has been shown that the use of Lévy flights reduces the maximum number of iterations required by approximately 10^4^ times compared to the Gaussian distribution [24].

The integration of Lévy flights into metaheuristic algorithms became widespread with the Cuckoo Search (CS) proposed by Yang and Deb [7]. Yang and Deb [23] demonstrated that, compared to PSO and GA, CS exhibits superior convergence behavior in terms of the number of function evaluations. The effectiveness of Lévy flight arises from its more efficient search structure than the Gaussian-based random walk in environments where targets are sparsely and unpredictably distributed [25]. In the Flower Pollination Algorithm (FPA), the global pollination process is also modeled by Lévy flight [26]. In the integration of Lévy flight into the Firefly Algorithm (FA), the randomization term is sampled from the Lévy distribution, which increases the algorithm’s ability to escape local optima [22].

The effectiveness of Lévy flights is supported by biological observations and mathematical properties. Lee and Yao [25] proposed Fast Evolutionary Programming (FEP), which replaces Gaussian-based mutation with the Cauchy distribution, a special case of the Lévy distribution; on 14 benchmark functions—including three unimodal, six multimodal, and five multimodal functions with relatively few local minima—they obtained more robust results than standard evolutionary programming based on Gaussian and Cauchy mutation.

The extensive applications of Lévy flight in metaheuristic algorithms justify its use as an alternative randomization principle to the Gaussian distribution. This is particularly evident in classes of problems in which the target distribution is sparse, there is no prior knowledge of the search space, and local minima are prevalent [23].

Lévy flights are a powerful search primitive based on a heavy-tailed probability distribution that includes both rare large jumps and frequent short steps within the same distribution. The Cuckoo Search (CS) proposed by Yang and Deb [7] was one of the first metaheuristic algorithms to use steps sampled from this distribution as its exploration mechanism. In CS, a Lévy step is applied in the global search space in a scale-free manner; that is, the direction and magnitude of the step are independent of the current position of the search agent. Although this approach is effective for scanning the search space on a broad scale, it also carries the risk of generating unnecessarily large steps during the convergence phase. Reynolds and Frye [27] demonstrated that Lévy flights are observed not only in insects but also in human eye movements and the foraging patterns of predatory animals, reinforcing the biological universality of this distribution.

Another recent important study by Zhong et al. [28] proposed a robust metaheuristic swarm intelligence algorithm called Beluga Whale Optimization (BWO), which is based on the complex social behaviors of beluga whales in nature, their synchronized swimming strategies, and the ecological cycles known as “whale fall.” The Lévy flight mechanism integrated into the algorithm enables high mobility in the search space through long-range steps that are suddenly added to short-range, precise explorations. Thus, the BWO is prevented from getting trapped in deceptive local optima and can converge rapidly and stably toward the global best solution. Other notable algorithms based on Lévy flight include Lévy Flight Ant Colony Optimization [29] and the Fractal Search Algorithm [30].

### 2.4. Preservation of Population Diversity and Operator Selection

Although the primary purpose of advanced operators such as Lévy flights and chaotic maps is to prevent algorithm stagnation, the active preservation of population diversity remains one of the most important research topics in the metaheuristic literature. Premature convergence, namely, the population becoming trapped in a local optimum, is a frequently encountered problem, especially in multimodal and rotated functions.

The main approaches to this problem can be classified as follows: (i) multi-population architectures (island model), (ii) memory-based adaptation, and (iii) diversification of learning strategies. Comprehensive learning-based particle swarm optimization (CLPSO) [31] increases personal best (pbest) diversity by allowing each particle to learn from the knowledge of different individuals across different dimensions. In contrast, the adaptive Differential Evolution algorithm JADE [32] adapts the F and CR values online by recording successful parameter values and updating them through a Cauchy-based mutation factor adaptation and a normal-distribution-based crossover adaptation.

In both approaches, the diversity criterion is used as an indirect input for parameter adaptation; the loss of diversity does not directly trigger an action decision. This gap paves the way for design approaches that directly link the diversity criterion to an operator-selection mechanism. This issue is examined in detail as one of the original contributions of the proposed method, which aims to make the optimization process more intelligent and adaptive.

### 2.5. Spatial Optimization and AI in Precision Agriculture

A parallel, largely separate literature has developed around the use of artificial intelligence and metaheuristic search for spatial decision problems in precision agriculture, most directly relevant to the real-world monitoring-network placement application evaluated in this study. Wireless Sensor Network (WSN) and monitoring-station deployment is a canonical example: sensor or trap nodes must be positioned within a field to maximize coverage and detection reliability under a limited node budget and energy or logistics constraints, a problem structurally identical to the maximal covering location formulation used in this study [33]. Ferentinos and Tsiligiridis [34] were among the first to apply a genetic algorithm to this class of problem, explicitly using a precision agriculture sensor deployment as their motivating case. Jawad et al. [35] provide a broader review of energy-efficient WSN design for precision agriculture, cataloguing subsequent metaheuristic (including GA- and PSO-based) placement and routing strategies. More generally, particle-swarm-based node deployment continues to be developed outside agriculture specifically (e.g., [36]), underscoring that GA and PSO remain the de facto baseline algorithms against which new spatial-optimization metaheuristics, including SPO, are typically compared in this application domain.

A second, complementary strand concerns AI-driven pest and disease detection itself, rather than the placement of the sensing infrastructure. Convolutional neural networks and related deep learning architectures have achieved classification accuracies exceeding 95% and detection/segmentation precision above 90% for a range of plant diseases and insect pests from image data [37]. These detection systems and the spatial monitoring-network optimization problem addressed in this paper are complementary rather than competing components of an integrated pest management pipeline: detection models determine what is present at a given location, while placement optimization determines where monitoring capacity should be concentrated in the first instance so that detection can occur early enough to inform an economically meaningful intervention decision.

Beyond sensor and trap network deployment, metaheuristic algorithms are increasingly applied to other spatially and temporally complex precision agriculture tasks. Unmanned Aerial Vehicle (UAV)-based pesticide application is one such area: Faiçal et al. [38] developed a genetic-algorithm-based system that adapts UAV spray-route correction intensity to real-time wind conditions, improving spraying accuracy over static flight-control policies. Precision irrigation scheduling has followed a parallel trajectory: Alonso Campos et al. [39] used a parallel multi-objective genetic algorithm to optimize real-time energy consumption and valve-switching sequences across a pressurized irrigation network. These applications, together with the monitoring-network placement problem addressed in Section 4.8, illustrate a common pattern in contemporary precision agriculture: as agronomic interventions become more spatially and temporally granular, the underlying decision problems increasingly inherit a combinatorial complexity that favors population-based metaheuristic search over static or manually designed rules.

## 3. Proposed Metaheuristic Method: Sunn Pest Optimization (SPO)

### 3.1. Biological Background: Eurygaster Integriceps

The Sunn pest, *Eurygaster integriceps* Puton, 1881 (Hemiptera: Scutelleridae), is one of the most economically significant cereal pests in Turkey, Iran, Iraq, Syria, and the Caucasus, where annual grain yield and quality losses can reach 70% in heavily infested regions [40,41]. The species completes a single generation per year and passes through four behaviorally distinct phases: (i) overwintering diapause, (ii) spring migration and field colonization, (iii) intensive feeding and reproduction, and (iv) autumn migration and return to the overwintering site. Each phase supplies a distinct functional property that is abstracted into the corresponding algorithmic component of the SPO. These mappings are summarized in Table 1 and elaborated upon in the following subsections.

#### 3.1.1. Seasonal Vertical Migration and Phase Separation

Adult Sunn pests overwinter at altitudes of 800–2000 m in oak (*Quercus* spp.) and beech (Fagus orientalis) forests beneath leaf litter. When the ambient temperature exceeds approximately 15 °C in spring, the population descends en masse to lowland wheat fields in a dispersal movement that spans several days, covers large distances, and lacks a fixed directional target [40]. The resulting transition is from a spatially diffuse overwintering configuration to one concentrated around a resource patch (the wheat field) in the spring.

These two phases—broad-area, undirected dispersal followed by resource-centered aggregation—define the functional distinction between the global search and local refinement that underpins the SPO phase-separated update rule. The heavy-tailed character of the spring migration, in which most individuals travel short distances while a minority undertake very long-range displacements, motivated the use of a Lévy-distributed step in the exploration operator. Migration and settlement collectively occupy approximately 30–40% of the total life cycle [42], which motivated the default value chosen for the exploration ratio parameter.

#### 3.1.2. Intra-Field Group Foraging and Differential Movement

Once established in the field, Sunn pest populations feed on dense aggregations rather than isolated individuals. This pattern is consistent with chemically mediated conspecific attraction: individuals produce sex pheromones (Buleza and Mikheev, 1979, as cited in [43]), and this chemical communication may underlie the tendency to aggregate around feeding patches. This behaviour has two functional consequences relevant to the exploitation operator. First, individuals move toward the highest-quality patch, motivating a social attraction component directed at the best current solution. Second, an individual’s trajectory is shaped by the relative positions of nearby conspecifics, not solely by the global best. This second property motivates a differential component that encodes a local population density gradient and guides movement without requiring explicit gradient computation.

#### 3.1.3. Population-Level Elimination: Carabidae Predator Pressure

Carabidae ground beetles are natural predators of Sunn pest nymphs and adults within the field. Their predatory activity is random in target selection but disproportionately affects individuals that are poorly positioned, such as those distant from the feeding resource or otherwise exposed. Consequently, the lowest-fitness segment of the population faces an elevated risk of elimination [40]. This ecological mechanism motivates two design decisions in the Carabid operator:

- Elimination pressure is concentrated on below-average individuals rather than applied uniformly across the population, reflecting the selective nature of predation in this species.
- A predated individual is replaced by a completely new, randomly initialized individual, reflecting the ecological reality that the vacated niche is filled by a newcomer with no spatial relationship to its predecessor.

#### 3.1.4. Individual Selective Pressure: Beauveria Bassiana Infection

Beauveria bassiana, an entomopathogenic fungus, is a natural population regulator of *E. integriceps* [40]. Critically, infection does not immediately kill the host; instead, it first compromises locomotor organs—legs and wing muscles—while the individual remains alive. If the immune response overcomes the infection, the individual recovers full mobility; otherwise, mortality follows. This partial, organ-level, and potentially reversible selectivity motivates two defining features of the Beauveria operator:

- Only the dimensions most deviant from the best-known solution (“diseased organs”) are modified, rather than reinitializing the entire position vector.
- The modified configuration is accepted only if it improves the fitness; otherwise, the individual “recovers” and retains its previous position. This conditional acceptance is the property that distinguishes the Beauveria operator from the unconditional restart logic of the Carabid and Parasitoid operators.

#### 3.1.5. Collective Egg Destruction: Trissolcus Parasitoid Pressure

Parasitoid wasps of the genus Trissolcus oviposit into Sunn pest egg masses, destroying entire clutches (approximately 10–15 eggs per mass). The parasitization rate varies dramatically from year to year—from approximately 20% to 80% of the population—and is density-dependent: dense, homogeneous egg clusters are more easily located and exploited by Parasitoids [44]. Therefore, this pressure is collective, sudden, and spatially concentrated. The Parasitoid operator encodes this in two ways:

- The operator simultaneously re-initializes a substantial fraction of the population, which is larger than any other operator in the hierarchy, reflecting the collective scale of egg mass destruction.
- The density-dependence of real Parasitoid pressure motivates an additional trigger condition: beyond the periodic stagnation-based activation shared by all operators, the Parasitoid operator is also triggered when the population’s spatial diversity falls below a threshold, irrespective of the stagnation count. Denser, more homogeneous populations are more vulnerable in nature; correspondingly, a spatially crowded population in the algorithm triggers an immediate, large-scale re-initialization.

#### 3.1.6. Inter-Generational Memory: Return to the Overwintering Site

In autumn, adults that return to the overwintering site are not a random subset of the summer population. Those that fed most successfully—accumulating sufficient fat reserves—show higher overwintering survival and greater reproductive capacity in the following spring [40]. Therefore, the surviving cohort constitutes a small but high-quality founding population for the next season. This inter-generational memory motivates the elitism mechanism: the best-performing individuals are preserved across iterations, but the ratio is deliberately kept low. Only a small fraction of the population successfully overwinters in nature; a high elitism ratio would reduce diversity and counteract the spatial diversity trigger.

Table 1 consolidates all biological features, their functional rationale, and algorithmic counterparts.

### 3.2. Mathematical Model and Notation

This section presents the mathematical model and algorithmic details of the SPO. The SPO operates on a population of N individuals X = $\left\{X_{1},\ldots ,X_{N}\right\}$⊂R⌃D over T iterations. X_i_(t) ∈R⌃D denotes the position vector of individual i at iteration t; f: R⌃D →R is the objective function to be minimized; f_i_t_i_(t) = f(X_i_(t)) is the corresponding fitness value; and X_best_(t) is the best position observed up to iteration t, that is, argmin_i_ f(X_i_(t)). The vectors lb and ub define the search bounds, and the clipping operation X_i_(t + 1) ← clip(X_i_(t + 1), lb, ub) is applied after every update; this step is implicit in all equations below. The algorithm comprises three sequential processing stages: (i) phase-separated migration mechanism, (ii) tiered stagnation operator hierarchy, and (iii) elitism. Table 2 provides a consolidated view of the biological–algorithmic analogies and their equation references.

The initial population is generated by uniform random sampling within the search bounds, formalized as Equation (1)

(1) $$X_{i}\left(0\right)∼U\left(lb,ub\right),\;i=1,\ldots ,N$$

Here $U\left(lb,ub\right)$ denotes the *D*-dimensional continuous uniform distribution over the search bounds. Fitness values $fit_{i}\left(0\right)=f\left(X_{i}\left(0\right)\right)$ are computed for all individuals. $X_{best}\left(0\right)$ is initialized as $X_{\arg\min_{i}fit_{i}\left(0\right)}$.

### 3.3. Wind Coefficient

Both migration phases share a common wind coefficient that decays linearly over the course of the run as Equation (2):

(2) $$w\left(t\right)=w_{max}-t\cdot \left(\frac{w_{max}-w_{min}}{T}\right)$$

where w(t) is the wind coefficient at iteration t, w_max_ and w_min_ are the initial (maximum) and final (minimum) wind intensities, respectively, and T is the maximum iteration count. The default values were $w_{\max}=0.9$ and $w_{\min}=0.2$. The coefficient serves the same purpose as the inertia weight in PSO [45]. It allows for large steps with high exploration capacity early in the run, transitioning to a fine-grained local search as t→T.

### 3.4. Migration Mechanism

The core phase-separated update rule of SPO is partitioned into two phases according to the condition t<exploration ratio·T.

#### 3.4.1. Exploration Phase—Directional Lévy Flight

The exploration phase is active when t<explorationratio·T (default: t<0.4T), modelling the large-scale and unpredictable vertical migration of Sunn pests from overwintering sites to wheat fields. The position of each individual is updated as Equation (3):

(3) $$X_{i}\left(t+1\right)=X_{i}\left(t\right)+s\cdot L\left(\beta \right)⊙\left(X_{i}\left(t\right)-X_{best}\left(t\right)\right)+0.3\cdot w\left(t\right)\cdot r_{1}⊙\left(X_{best}\left(t\right)-X_{i}\left(t\right)\right)$$

where s=levy_scale=0.01 is a step-size scaling constant, and L(β) ∈R⌃D is a Lévy step vector generated via the Mantegna algorithm (Equations (4) and (5)); w(t) is the wind coefficient (Equation (2)); $r_{1}$∈ [0, 1]⌃D is a dimension-wise uniform random vector; and ⊙ denotes the Hadamard product.

The Lévy step vector L(β) is produced using the Mantegna algorithm [7,46]:

(4) $$L\left(\beta \right)=\frac{U}{{\left|V\right|}^{1/\beta }},\;U∼N\left(0,\sigma_{u}^{2}\right),\;V∼N\left(0,1\right)$$

(5) $$\sigma_{u}={\left[\frac{\Gamma \left(1+\beta \right)\cdot sin\left(\left(\pi \beta /2\right)\right)}{\Gamma \left(\left(1+\beta \right)/2\right)\cdot \beta \cdot 2^{\left(\beta -1\right)/2}}\right]}^{1/\beta }$$

where Γ is the gamma function, and β = 1.5 is the Lévy tail index. The first term of Equation (3), s·L(β)⊙(X_i_ – X_best_), generates heavy-tailed displacements scaled by the individual’s distance from the current best solution: an individual far from the leader produces large steps, whereas one nearby produces small steps. The rare but large jumps inherent in the Lévy distribution prevent premature entrapment in local optima [23,24]. The second term, 0.3·w(t)·r_1_⊙(X_best_ – X_i_), provides a weak attractor toward the best-known solution, preventing the search from degenerating into a pure random walk.

The directional structure of the Lévy step in SPO—L(β)⊙(X_i_ – X_best_)—differs from that in the standard Cuckoo Search [7], in which the step is applied without reference to the current leader. In SPO, this term moves the individual away from the leader with a magnitude proportional to their distance, producing a divergent exploration geometry that encourages broader coverage of the search space during the migration phase.

#### 3.4.2. Exploitation Phase—Differential Swarm Migration

When t≥explore_ratio·T, the update rule switches to the exploitation phase, modelling the resource-oriented, socially informed intra-field foraging behavior described as Equation (6).

(6) $$X_{i}\left(t+1\right)=X_{i}\left(t\right)+\alpha_{1}\left(t\right)\cdot r_{1}⊙\left(X_{best}\left(t\right)-X_{i}\left(t\right)\right)+\alpha_{2}\cdot r_{2}⊙\left(X_{r1}\left(t\right)-X_{r2}\left(t\right)\right)$$

where α_1_(t) = alpha_1_max_·w(t) is the time-varying social coefficient (alpha_1_max_= 1.5); α_2_ = 0.5 is the fixed differential coefficient; r_1_, r_2_∈ [0, 1] are independent random vectors; and X_r1_, X_r2_ are the positions of two individuals drawn independently at random from the population (i.e., r_1_ and r_2_ index individuals, not dimensions; i = r_1_ = r_2_ is permitted, in which case the differential term vanishes and the update reduces to the social term alone).

The social term α_1_(t)·r_1_⊙(X_best_ – X_i_) provides convergence pressure toward the best-known solution, whereas the differential term α_2_·r_2_⊙(X_r1_ – X_r2_) encodes relative directional information from the current population. This information is particularly valuable for rotated and ill-conditioned functions, where the axis-aligned update direction of a pure social term may be misaligned with the true descent direction [23]. The ratio α_1_max_:α_2_ = 1.5:0.5 (3:1) was calibrated empirically on a CEC 2017 subset; the sensitivity of this ratio and a self-adaptive variant are deferred to future work.

Equation (6) is structurally close to the DE/current-to-best/1 mutation strategy [4], $V_{i}=X_{i}+F_{1}\cdot \left(X_{best}-X_{i}\right)+F_{2}\cdot \left(X_{r1}-X_{r2}\right)$, rather than to DE/best/1 or DE/rand/1: both formulations add a best-attraction term and a differential term to the current position, instead of replacing it outright. Four differences nonetheless distinguish SPO’s exploitation update from a direct application of DE/current-to-best/1. First, DE/current-to-best/1 generates a trial vector that is accepted only under an explicit, per-individual greedy selection step, whereas SPO applies Equation (6) unconditionally to every individual every iteration, relying on the stagnation operator hierarchy rather than per-update selection to correct individuals that worsen. Second, the coefficients $F_{1}$, $F_{2}$ in DE/current-to-best/1 are typically fixed, or, in adaptive variants such as L-SHADE, self-adapted via a success-history memory as uniform scalars, whereas SPO’s $\alpha_{1}\left(t\right)$ decays deterministically over the run through the wind coefficient (Equation (2)), and $r_{1}$, $r_{2}$ are dimension-wise random vectors rather than scalars. Third, Equation (6) is active only during the exploitation phase (*t* ≥ explore_ratio·*T*), preceded by a structurally distinct Lévy flight exploration phase (Equation (3)) that has no counterpart in a single-strategy DE variant. Fourth, SPO’s population-level diversity trigger (Equations (7)–(9)) and tiered stagnation hierarchy operate independently of, and on top of, the per-individual update rule, providing a second, population-level mechanism for escaping stagnation that DE/current-to-best/1 does not possess in its standard form. This structural proximity to DE/current-to-best/1, markedly closer than to DE/best/1, underscores that SPO’s exploitation phase is best understood as a variant within the differential evolution family of update rules rather than a wholly novel operator; SPO’s original contributions, as stated in Section 1, reside in the phase separation, the stagnation hierarchy, and the diversity-override mechanism, rather than in the exploitation update rule considered in isolation. This relationship also motivated the inclusion of L-SHADE [47]—a state-of-the-art, self-adaptive descendant of the same current-to-best/1 lineage—as an explicit baseline in Section 4.5.

### 3.5. Tiered Stagnation Hierarchy and Spatial Diversity

#### 3.5.1. Stagnation Counter

SPO manages the three ecological pressure operators through a single integer counter stag ∈$N_{0}$, which is updated at each iteration as Equation (7):

(7) $$stag\left(t\right)=\left\{\begin{array}{cc} 0, & if\;f_{best}\left(t\right)<f_{best}\left(t-1\right)-10^{-10}\\ stag\left(t-1\right)+1, & otherwise \end{array}\right.$$

where $f_{best}\left(t\right)={min}_{i}\left(f\right)it_{i}\left(t\right)$ is the population-best fitness at iteration t. The tolerance of 10^−10^ prevents spurious improvements caused by floating-point noise from resetting the counter.

#### 3.5.2. Spatial Diversity Measure and Diversity-Override

On rotated functions with high condition numbers, the population can cluster in a narrow region of the search space before the stag reaches its critical thresholds. To detect this independently, the mean Euclidean distance to the population centroid, denoted as Δ(t) in Equation (8), is computed at each iteration:

(8) $$\Delta \left(t\right)=\frac{1}{N}\sum_{i=1}^{N}{∥X_{i}\left(t\right)-\mu \left(t\right)∥}_{2},\;\mu \left(t\right)=\frac{1}{N}\sum_{i=1}^{N}X_{i}\left(t\right)$$

A diversity threshold is established relative to the initial diversity Δ(0) computed at t = 0 as Equation (9):

(9) $$\mathrm{div}\_\mathrm{threshold}=\max\left(\Delta \left(0\right)\cdot \varepsilon ,10^{-10}\right),\;\mathrm{where}\;\varepsilon =10^{-4}$$

When Δ(t) falls below this threshold, the stagnation counter is immediately forced to the Parasitoid activation level, irrespective of the number of iterations elapsed without improvement. While population diversity serves as an adaptive triggering signal in other metaheuristics, SPO distinctly couples this signal directly to operator selection, bypassing the stagnation counter entirely within its three-tier hierarchy. Because Δ(0) is computed from *N* i.i.d. uniform samples, the law of large numbers ensures convergence to its theoretical expectation with low variance. Consequently, the threshold variation across independent runs is negligible, ensuring stable operator triggering. This stability is empirically reflected in the low standard deviations and consistent run-to-run performance.

#### 3.5.3. Carabid Operator

When the stagnation counter reaches the Carabid threshold (Equations (10) and (11)), the worst-performing 20% of individuals drawn from the above-average-cost subset are randomly re-initialized.

(10) $$W=\left\{i:fit_{i}\left(t\right)>\frac{1}{N}\sum_{j=1}^{N}fit_{j}\left(t\right)\right\},\;\left|K\right|=\left\lceil \left|W\right|\cdot 0.2\right\rceil$$

(11) $$X_{i}\left(t+1\right)∼U\left(lb,ub\right)\;\forall i\in K,\;fit_{i}\left(t+1\right)=f\left(X_{i}\left(t+1\right)\right)$$

*K* represents the subset of the weakest 20% of individuals from *W* selected for elimination and subsequent reinitialization. If W=∅ (i.e., all individuals share identical fitness, indicating complete convergence), the operator takes no action, as the population has reached a degenerate fixed point.

#### 3.5.4. Beauveria Operator

When the stagnation counter reaches the Beauveria threshold, the n^*d*^ = ⌊0.3D⌋ dimensions most deviant from X_best_ are identified for each individual, and those dimensions are replaced with X_best_-centred Gaussian perturbations (Equations (12)–(14)):

(12) $$J_{i}={argTop}_{n_{d}}\left(\left(\left|X_{i,d}\left(t\right)-X_{best,d}\left(t\right)\right|\right)\right),\;d\in \left\{1,2,\ldots ,D\right\}$$

(13) $${\hat{X}}_{i,d}\left(t\right)=X_{best,d}\left(t\right)+N\left(0,0.01\cdot \left(ub_{d}-lb_{d}\right)\right),\;\forall d\in J_{i}$$

(14) $$X_{i}\left(t+1\right)=\left\{\begin{array}{cc} {\hat{X}}_{i}\left(t\right), & if\;f\left({\hat{X}}_{i}\left(t\right)\right)<fit_{i}\left(t\right)\\ X_{i}\left(t\right), & otherwise \end{array}\right.$$

$J_{i}$ is the subset of dimension indices where the *i*-th individual exhibits the maximum absolute deviation from the global best $X_{best}$; ${argTop}_{n_{d}}$ is an operator that returns the indices of the largest $n_{d}$ values from a given set, effectively targeting the most unsuccessful parameters of the solution vector; $n_{d}$ is the specific number of dimensions to be mutated, determined by the ratio parameter of the operator ($n_{d}=\left\lceil D\cdot beauveria\_dim\_ratio\right\rceil$); ${\hat{X}}_{i}\left(t\right)$ is the candidate (mutated) position vector generated for the *i*-th individual; $ub_{d}$ and $lb_{d}$ are the upper and lower search boundaries of the *d*-th dimension, respectively; $N\left(0,\ldots \right)$ is the Gaussian noise injected to facilitate dimensional local search and maintain population diversity around the elite solution.

Equation (14) represents a strict greedy selection mechanism, ensuring that the mutated candidate ${\hat{X}}_{i}\left(t\right)$ replaces the original individual only if it yields a superior fitness value. The conditional acceptance in Equation (14) makes the Beauveria operator the only member of the stagnation hierarchy that incorporates elitist selection within its own logic, reflecting the biological scenario in which recovery is possible: the modified configuration is accepted only if it constitutes a genuine improvement. The Carabid and Parasitoid operators, by contrast, model complete replacement of an individual and therefore apply unconditionally.

#### 3.5.5. Parasitoid Operator

When the stagnation counter reaches the Parasitoid threshold (Equations (15)–(17)), or when the diversity-override condition Δ(t)< div_threshold is met, 30% of the population is re-initialized at random (excluding the current best individual i*):

(15) $$i^{*}={argmin}_{i}\left(f\right)it_{i}\left(t\right),\;K^{\prime }\subset \left\{1,\ldots ,N\right\}\setminus \left\{i^{*}\right\},\;\left|K^{\prime }\right|=\left\lfloor \left(N-1\right)\cdot 0.3\right\rfloor$$

(16) $$X_{i}\left(t+1\right)∼U\left(lb,ub\right)\;\forall i\in K^{\prime },\;fit_{i}\left(t+1\right)=f\left(X_{i}\left(t+1\right)\right)$$

(17) $$X_{i^{*}}\left(t+1\right)=X_{i^{*}}\left(t\right)$$

$i^{*}$ is the index of the current global best individual (the elite) in the population at iteration *t*. $K^{\prime }$ is a randomly selected subset of indices representing the individuals targeted by the Parasitoid attack. The elite individual $i^{*}$ is strictly excluded from this set. $\left|K^{\prime }\right|$ is the cardinality of the subset $K^{\prime }$, determined by the predefined kill ratio (0.3 or 30%) applied to the remaining non-elite population (N−1); $U\left(lb,ub\right)$ is continuous uniform distribution across the lower and upper boundaries of the search space. This operation scatters the targeted individuals to completely new random positions, injecting massive spatial diversity to escape local optima. Equation (17) is a strict elitism mechanism ensuring that the most successful solution is immune to the Parasitoid pressure and is preserved identically for the subsequent generation.

After the operator executes, the stagnation counter is reset: stag ← 0. This reset applies regardless of whether the trigger was the counter threshold or the diversity-override condition.

#### 3.5.6. Rationale for the Stagnation Thresholds

The three activation thresholds form an arithmetic progression with a common spacing of eight iterations. This ensures that each successive operator receives an equally long uninterrupted window before a more disruptive intervention becomes eligible. Because these conditions are evaluated independently rather than as mutually exclusive alternatives, reaching a higher threshold causes its operator to execute alongside any eligible lower-tier operators. Consequently, the algorithmic response accumulates in severity rather than abruptly switching strategies.

This uniform spacing is driven by biological design rather than numerical optimization. It mirrors the graduated severity of the corresponding ecological pressures (localized predation, organ-level infection, then population-scale parasitism). The mildest operator (Carabid) acts first, providing adequate time for targeted recovery before the algorithm escalates to the most disruptive operator (Parasitoid). While halving or doubling the spacing (e.g., 4/8/12 or 16/32/48) would compress or dilate this escalation schedule, it would preserve the qualitative structure of the hierarchy. Unlike the explore_ratio and population size *N*, these threshold values were not subjected to a systematic sensitivity sweep. Optimizing this spacing and exploring unequal intervals remain areas for future investigation.

### 3.6. Elitism (Overwintering Memory)

At the end of each iteration, the worst-performing 10% of individuals are replaced by copies of the corresponding elite individuals, as shown in Equation (18):

(18) $$X_{worst\left(k\right)}\left(t+1\right)\leftarrow X_{elite\left(k\right)}\left(t+1\right),\;fit_{worst\left(k\right)}\left(t+1\right)\leftarrow fit_{elite\left(k\right)}\left(t+1\right)$$

*N* is the total population size. $\left\lfloor 0.1\cdot N\right\rfloor$ is the number of elite individuals selected for preservation, corresponding to the top 10% of the population. The floor function ensures an integer count. The indices of the *k*-th best-performing and *k*-th worst-performing individuals are denoted by elite(k) and worst(k), respectively. Equation (18) represents the biological “wintering memory” (elitism) phase. By directly overwriting the state and fitness of the weakest individuals with those of the strongest elites, the algorithm guarantees that high-quality solutions are preserved across generations and prevents regression in the convergence curve.

Crucially, these copied individuals are not frozen in place; they fully participate in the migration mechanism during the subsequent iteration. The low elite ratio ensures that most of the population retains full diversity, while any spatial clustering induced by the elitist copies is naturally counteracted by the diversity-override trigger.

### 3.7. Pseudocode

Algorithm 1 presents the complete SPO pseudocode.
**Algorithm 1** Sunn Pest Optimization (SPO)**Require:** f(x), *D*, lb, ub, *N*, *T*, all parameters **Ensure:** $X_{best}$, $f_{best}$  1:$X_{i}∼U\left(lb,ub\right)$, i=1…N               ▹ Initial population  2:$fit_{i}\leftarrow f\left(X_{i}\right)\;\forall i$  3:$X_{best}\leftarrow X_{\arg\min_{i}fit_{i}}$, $f_{best}\leftarrow \min_{i}fit_{i}$  4:$\Delta \left(0\right)\leftarrow \frac{1}{N}\sum_{i}{∥X_{i}-\mu \left(0\right)∥}_{2}$              ▹ Equation (8), t=0  5:$div\_threshold\leftarrow \max(\Delta \left(0\right)\cdot \varepsilon ,10^{-10})$          ▹ Equation (9)  6:stag←0, $f_{prev}\leftarrow f_{best}$  7:**for** t=0 **to** T−1 **do**  8:      $w\left(t\right)\leftarrow w_{max}-t\cdot \left(w_{max}-w_{min}\right)/T$          ▹ Equation (2)  9:      **if** t<0.4T **then**10:            X← LevyMigration(*X*, $X_{best}$, w(t))       ▹ Equations (3)–(5)11:      **else**12:            X← DifferentialMigration(*X*, $X_{best}$, w(t))       ▹ Equation (6)13:      **end if**14:      $fit_{i}\leftarrow f\left(X_{i}\right)\;\forall i$; update $X_{best}$, $f_{best}$15:      $\Delta \left(t\right)\leftarrow \frac{1}{N}\sum_{i}{∥X_{i}-\mu \left(t\right)∥}_{2}$              ▹ Equation (8)16:      $stag\leftarrow (f_{best}<f_{prev}-10^{-10})?0:stag+1$          ▹ Equation (7)17:      **if** Δ(t)<div_threshold **then**18:            stag←max(stag,24)              ▹ diversity-override19:      **end if**20:       **if** stag≥8 **then**21:            [X,fit]← Carabid(X,fit)          ▹ Equations (10) and (11)22:      **end if**23:      **if** stag≥16 **then**24:            [X,fit]← Beauveria($X,fit,X_{best}$)       ▹ Equations (12)–(14)25:      **end if**26:      **if** stag≥24 **then**27:            [X,fit]← Parasitoid(X,fit); stag←0     ▹ Equations (15)–(17)28:      **end if**29:      Elitism: worst 0.1N← best 0.1N             ▹ Equation (18)30:       $f_{prev}\leftarrow f_{best}$31:**end for**32:**return** $X_{best}$, $f_{best}$

### 3.8. Computational Complexity

The migration mechanism incurs O(N·D) time per iteration. The spatial diversity computation (Equation (8)) adds an additional O(N·D) operations. The Carabid and Parasitoid operators require O(K·D) (where K≪N). However, the Beauveria operator introduces an O(N·DlogD) cost: identifying the $n_{d}$ most-deviant dimensions for a given individual (Equation (12), ${\mathrm{argTop}}_{n_{d}}$) requires sorting that individual’s *D*-length deviation vector via a standard comparison sort (implemented with argsort). Because this is repeated independently for each of the *N* individuals whenever the operator activates, the dominant per-iteration complexity becomes O(N·DlogD). Combined with the O(NlogN) elitism step, the absolute worst-case total complexity over the full run is O(T·N·DlogD). While this introduces an O(logD) factor above classical baselines like PSO, the Beauveria operator strictly executes only when stag≥16, significantly diluting its contribution to the aggregate runtime.

The practical magnitude of this O(DlogD) overhead depends heavily on dimensionality. At D=10, the sorting cost is mathematically trivial ($10\log_{2}10\approx 33$ operations versus 10 for standard migration), rendering the overhead negligible. However, because the argsort term scales logarithmically while the rest of the update scales linearly, its relative share of the per-iteration cost grows proportionally with *D*, presenting a theoretical scalability boundary at higher dimensions (Section 4.9). In the present experiments, this specific asymptotic growth is masked in the wall-clock runtimes because the $O(D^{2})$ evaluation cost of the CEC benchmark shift-and-rotation matrices heavily dominates the O(DlogD) operator overhead. Disentangling the operator’s isolated runtime contribution from the benchmark’s quadratic evaluation cost requires dedicated profiling across a wider range of *D*, which remains a direction for future work.

### 3.9. Fitness Evaluation Cost Analysis

Several methods have been proposed in the literature for quantifying the fitness evaluation (FE) cost of metaheuristic algorithms [48,49,50,51]. This study builds upon the recently proposed methodology introduced by Sari and Yildirim [52], which enhances the traditional Big-O notation by incorporating a direct count of objective function evaluations. In their general form (their Equation (5)), the total FE cost is expressed as an initial-population term plus a decomposition-weighted, per-operator sum accrued over iterations and population members, plus an optional weighting-strategy term. Under this framework, the decomposition multiplier is set to M = 1 for every algorithm considered here, as none of them—SPO included—partitions the decision vector into subcomponents (their Equation (2) reduces to their Equation (1) in this case), and no additional weighting-strategy term is applied, so only the initialization term and the operator-level term (their Equation (3)/Equation (4)) are retained.

The baseline algorithms used for comparison—GA, PSO, GWO, WOA, and DE in their standard formulations—do not incorporate sub-decomposition or nested local search procedures; therefore, their per-iteration FE costs are identical and equal to the population size N. In Sari and Yildirim’s notation, this corresponds to a single evaluation per individual per iteration (N_*S*_ + N_*Cr*_ + N_*M*_ = 1), summed over N_*I*_ = T iterations and N_*P*_ = N individuals, plus one evaluation per individual for the initial population (their initial-population term with N_*neighbor*_ = 1), which together reproduce the N + (T·N) formula below. SPO shares this baseline cost for its migration phase; however, owing to its dynamic operator hierarchy, it incurs additional evaluations whenever the Carabid, Beauveria, or Parasitoid operators are triggered. Because these operators are stagnation-triggered rather than executed at every iteration, we express their contribution as a closed-form function of operator activation counts (K, A, P) rather than as a raw double sum over all (iteration, individual) pairs; this is algebraically equivalent to evaluating Sari and Yildirim’s operator-level term (Equation (3)) with N_*S*_ + N_*C*_*r* + N_*M*_ = 0 for individuals and iterations where no operator fires and a nonzero operator-specific count where one does, collapsed into per-activation totals for readability. Therefore, the total FE budget of the SPO is not fixed but depends on how frequently each operator is activated during a given run. Table 3 summarizes the FE cost structures for all the algorithms.

In this table, N: population size; T: maximum iterations; K, A, P: number of activations of the Carabid, Beauveria, and Parasitoid operators, respectively; Σ(K,A,P) = (K·N·0.20) + (A·N) + (P·N·0.30); T_*G*_: the (smaller) iteration count used for GBFIO, reduced relative to T so that its higher per-iteration evaluation cost (28 evaluations per individual, reflecting its three internal search stages) still yields the same total FE budget as the other algorithms; N_*P*_(*g*): L-SHADE’s generation-dependent population size; FE_*max*_: the shared total-FE budget, held equal to N + (T·N) for every algorithm in the comparison.

This structure makes it explicit that SPO consumes a standard per-iteration FE budget during its migration phase—identical to that of the baseline algorithms—while the stagnation operators introduce a run-dependent surplus. In the worst case, where all three operators activate at every possible opportunity, the surplus remains bounded by O(T·N), preserving the same asymptotic order as the migration cost itself. In practice, operator activations are infrequent by design (activation thresholds of 8, 16, and 24 stagnation iterations, respectively), so the empirical FE overhead relative to PSO or GA is modest in practice, consistent with the per-iteration wall-clock runtimes measured during the benchmark runs. GBFIO and L-SHADE depart from the fixed-N-per-iteration pattern of the six other algorithms—the former through a higher, but still fixed, per-individual evaluation count, the latter through a shrinking population size under generation—yet both remain fully expressible within the same FE-counting framework, and both were calibrated to consume the same total FE budget as the other six algorithms, which keeps the baseline FE budget equalized across the benchmark comparison, though SPO’s stagnation operators still add the run-dependent surplus described above on top of that shared baseline.

### 3.10. Algorithm Parameters

Table 4 lists all the SPO parameters, along with their default values, relevant equations, and biological counterparts. Parameter values were determined through a combination of biological analogy (e.g., the explore_ratio from life-cycle proportions; the elitism ratio from overwintering survival rates) and empirical calibration on a CEC 2017 subset (D = 10). It should be noted that this calibration subset overlaps with the function suite used for the final performance comparison, rather than constituting a fully disjoint calibration set; the implications of this overlap for the reported performance advantage are discussed explicitly as a limitation. Problem-specific tuning, self-adaptive variants, and calibration on an independent benchmark suite (e.g., CEC 2014 or a held-out functional subset) will be reserved for future studies.

## 4. Experimental Results and Discussion

### 4.1. Experimental Design

To evaluate the proposed method, SPO was benchmarked against seven diverse algorithms: GA, PSO, GWO, WOA, DE, GBFIO, and L-SHADE. All algorithms were evaluated under identical conditions: population size N = 30, maximum iterations T = 1000, and search bounds [–100, 100]^D^. The core comparison was conducted at D = 10, followed by a dedicated scalability analysis at D = 30. Each algorithm was executed for 30 independent runs per function, and the seed for run *i* was fixed at 100·i + 7 to ensure reproducibility. The reported performance metrics are mean fitness, standard deviation, and rank. Statistical significance was assessed using the two-sided Wilcoxon rank-sum test with Bonferroni correction for seven simultaneous comparisons ($\alpha^{\prime }=0.01/7$). All tests were conducted on a workstation equipped with an Intel Core i7-10750H CPU, and 16 GB RAM. Full per-function mean ± std tables for both benchmark suites and the complete parameter-sensitivity results are provided in Appendix A Table A1, Table A2, Table A3, Table A4, Table A5 and Table A6. The main text reports the consolidated ranking in Table 5 and the statistical comparisons on which the paper’s conclusions rest.

The test suite comprised two benchmark packages: CEC 2017 (F1–F10, shifted and rotated) and CEC 2022 (F1–F6, shifted and rotated). The CEC 2017 comprises unimodal (F1–F2) and multimodal (F3–F9) functions, as well as a hybrid composite function (F10). CEC 2022 covers the Zakharov, Rosenbrock, Dixon–Price, Rastrigin, Lévy, and composite functions. Pure Python (ver. 3.10.21)/NumPy implementations with deterministic shift vectors and rotation matrices were used throughout the study. These are original re-implementations of the classical shifted/rotated function definitions.

The comprehensive eight-algorithm comparison was conducted at D=10, since CEC 2022 specifies D∈{10,20} and CEC 2017 specifies D∈{10,30,50,100}, making D=10 the smallest standard setting shared by both suites and keeping the primary 8×16×30×1000-run comparison computationally tractable. The subsequent sections evaluate core performance and parameter sensitivity at D=10, followed by a scalability analysis at D=30 and a real-world agricultural monitoring application.

### 4.2. Main Benchmark Results

#### 4.2.1. CEC 2017

Appendix A Table A1 reports the mean fitness and standard deviation for each algorithm on the CEC 2017 functions (N = 30, explore_ratio = 0.4, T = 1000). Bold values denote the best mean for each function.

When evaluating the subset of classical baseline algorithms (GA, PSO, GWO, WOA, DE), SPO achieved the best mean on F4, though the state-of-the-art L-SHADE slightly edges past this result ($1.89\times 10^{1}$ vs. SPO’s $1.90\times 10^{1}$), while GBFIO trails the entire field on this function ($2.05\times 10^{1}$). DE achieves the best mean among the classical algorithms on F1, F2, F6, and F9. On these predominantly separable or near-convex landscapes, DE’s classical differential mutation converges to near-machine-precision solutions (e.g., $2.63\times 10^{-24}$ on F1 and $4.90\times 10^{-24}$ on F9). L-SHADE’s adaptive control pushes convergence further still, reaching exactly zero on F1 and $1.35\times 10^{-29}$ on F9, whereas GBFIO underperforms on both ($4.93\times 10^{-1}$ and $5.68\times 10^{3}$, respectively). On the Rosenbrock-type F5, GA ($4.60\times 10^{2}$) and SPO ($5.18\times 10^{2}$) are the two best-performing algorithms within this classical subset, both one to three orders of magnitude below PSO, GWO, WOA, and DE, with GA narrowly ahead of SPO. L-SHADE improves on GA’s result by two further orders of magnitude ($4.09\times 10^{0}$), while GBFIO ($7.90\times 10^{4}$) again ranks among the weakest. PSO outperforms SPO on F6, F8, and F9—functions characterized by narrow valleys and dense local optima. In these environments, the individual memory term ($p_{best}$) in PSO provides an axis-aligned tracking advantage that SPO’s differential swarm term does not replicate. GBFIO, lacking any comparable mechanism, ranks last of all eight algorithms on both F6 and F9. Overall, while L-SHADE’s adaptive architecture (Table 5) predictably dominates the benchmark, SPO demonstrates a clear and consistent advantage over the recently published GBFIO across F1, F2, F5, F6, and F9. This contrast confirms that SPO’s structured operator hierarchy yields a substantially more robust search mechanism than other contemporary, bio-inspired alternatives.

#### 4.2.2. CEC 2022

Appendix A Table A2 presents the results obtained for the CEC 2022 benchmark. Among the classical baseline algorithms, GA achieved the lowest mean error on five out of six functions, while PSO performed best on one (F5). SPO consistently ranked second within this classical subset across all six functions, demonstrating robust performance across the suite. When expanding the evaluation to the full eight-algorithm field, L-SHADE’s adaptive control dominates, achieving the best mean on every function by several orders of magnitude (e.g., $9.87\times 10^{-30}$ on F1 and $5.97\times 10^{-3}$ on F5, versus SPO’s $1.87\times 10^{0}$ and $2.19\times 10^{1}$, respectively). GBFIO’s performance, by contrast, varies sharply: it is the weakest algorithm on F1 ($5.73\times 10^{3}$) and among the weakest on F2 ($2.18\times 10^{5}$), yet it outperforms SPO on F5 ($1.68\times 10^{1}$ versus $2.19\times 10^{1}$), trailing only L-SHADE and PSO.

GA’s strong performance on the CEC 2022 suite is attributable to the narrower valley geometry of its shifted–rotated constructions, which favors algorithms with individual positional memory. L-SHADE overcomes these geometric challenges entirely through its success-history-based parameter adaptation. GBFIO’s erratic pattern—competitive on the flat-valley F4 and F5 but failing on F1 and F2—indicates a lack of consistent adaptability to this suite’s topology. SPO’s consistent second-place ranking within the classical subset arises primarily on the multimodal and flat-valley functions (Rosenbrock, Dixon–Price, composite), where its diversity-override mechanism activates most frequently. Against GBFIO specifically, this yields a mixed record on the CEC 2022 suite rather than uniform superiority, consistent with the pairwise significance tests reported in Table 5.

**Figure A1:**
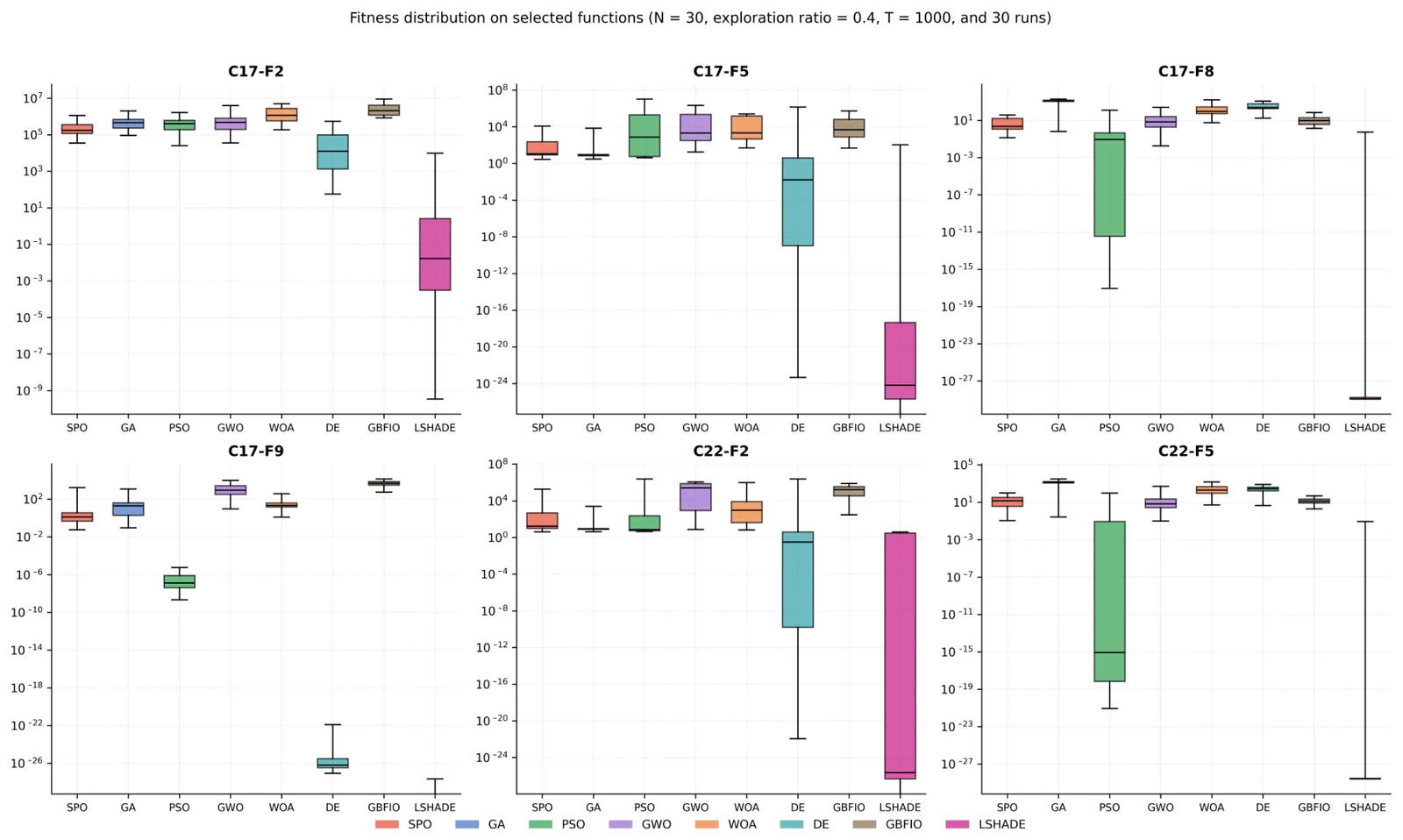
Fitness distribution on selected functions (N=30, exploration ratio =0.4, T=1000, and 30 runs).

Appendix A Figure A1 presents the fitness distributions for six representative functions drawn from the comprehensive comparison. The upper row highlights two functions where SPO performs strongly (C17-F2 and C17-F5) alongside one where PSO outperforms it (C17-F8). The lower row displays C17-F9, which PSO dominates, alongside two CEC 2022 functions (C22-F2 and C22-F5). The contrast between the narrow, low-valued interquartile ranges of SPO on C17-F2 and C17-F5 and the wide, high-valued ranges of PSO and GWO empirically illustrates the variance reduction achieved by SPO’s stagnation hierarchy. Conversely, on C17-F8 and C17-F9, the narrow PSO boxes at very low fitness values demonstrate the distinct advantage of individual positional memory on axis-aligned landscapes. This contrast clearly delineates the topological boundaries of SPO, pinpointing the specific geometric environments where its population-relative differential term falls short compared to algorithms equipped with explicit $p_{best}$ tracking.

### 4.3. Overall Ranking and Statistical Comparison

Table 5 consolidates the algorithmic performance across all 16 benchmark functions (10 from CEC 2017 and 6 from CEC 2022). The rightmost columns (“Sig. Better”, “Sig. Worse”, and “Tie”) summarize the two-sided Wilcoxon rank-sum test results with Bonferroni correction ($\alpha^{\prime }=0.01/7$), indicating the number of functions where SPO was statistically superior, inferior, or indistinguishable relative to the seven baseline algorithms.

SPO obtained the second-best average rank (3.62) across all 16 benchmark functions, trailing only L-SHADE (1.00). As a competition-winning algorithm, L-SHADE establishes the state-of-the-art upper bound on this suite. Among the remaining baselines, SPO demonstrates statistically significant superiority over WOA (ten better, four worse, two tied), GWO (eight better, zero worse, eight tied), and GBFIO (twelve better, one worse, three tied). Conversely, its performance against GA (six better, five worse, five tied) and DE (seven better, eight worse, one tied) is highly competitive but mixed, demonstrating that, while SPO ranks highly overall, it does not uniformly dominate these specific classical algorithms across all landscapes.

**Figure 1:**
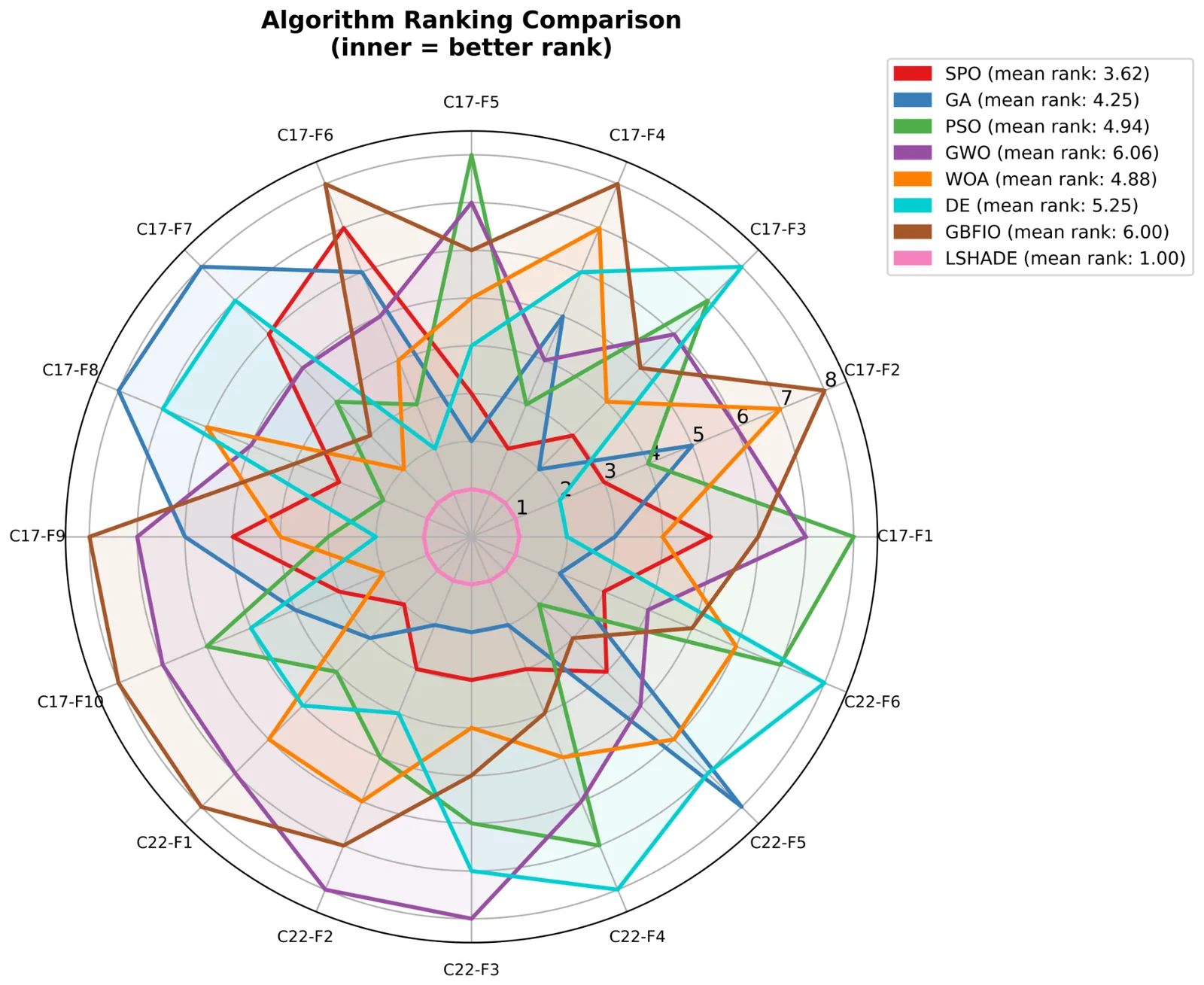
Normalized performance profile across all benchmark functions (N = 30, exploration ratio = 0.4, T = 1000, and 30 runs).

Figure 1 presents the per-function rank profile (rank 1, innermost; rank 8, outermost) for all eight algorithms. L-SHADE’s profile occupies the innermost ring on every function, visually confirming its dominance. SPO’s profile remains compact and inward on most functions, ranking second or third on F2, F3, F4, F5, F8, and F10 of CEC 2017, as well as F1 through F4 and F6 of CEC 2022. There are four exceptions where SPO falls to the outer half of the ranking: CEC 2017-F1 (rank 5) and F9 (rank 5), where DE’s near-machine-precision convergence on these separable landscapes outperforms the rest of the field (Appendix A Table A1); CEC 2017-F7 (rank 6), where WOA’s spiral-encircling mechanism is better suited to the deceptive, non-centroid-aligned optimum; and CEC 2017-F6 (rank 7), representing SPO’s weakest relative performance. Conversely, while GWO and GBFIO exhibit pronounced outward spikes on several multimodal functions, SPO maintains a notably tight and stable profile overall, reinforcing its structural robustness across diverse problem geometries.

### 4.4. Parameter-Sensitivity Analysis

Table A4 presents the population-size sensitivity analysis for SPO across the combined CEC 2017 and CEC 2022 suite at D=10. To contextualize the impact of varying *N*, SPO’s performance at each population size is ranked against the same seven baseline algorithms (GA, PSO, GWO, WOA, DE, GBFIO, and L-SHADE) evaluated in Section 4.5. At the default setting of N=30, SPO yields an average rank of 3.62, directly corresponding to the comprehensive baseline results established in Table 5. In a complementary analysis, the sensitivity of the exploration ratio is evaluated by comparing SPO’s internal mean fitness under varying configurations (0.3 versus 0.4), isolating the specific impact of this parameter on convergence behavior without external baseline rankings.

#### 4.4.1. Exploration Ratio

The exploration ratio determines the fraction of the iteration budget allocated to the Lévy flight exploration phase. Its default value of 0.4 was derived from the biological observation that *E. integriceps* devotes approximately 30–40% of its life cycle to migration and settlement (Section 3.1.1). To evaluate SPO’s sensitivity to this parameter, experiments were conducted using exploration ratios of 0.3 and 0.4, with N=30 and T=1000 held constant (Appendix A Table A3).

An exploration ratio of 0.3 yielded superior mean fitness on 10 of the 16 functions, whereas a ratio of 0.4 performed better on the remaining six (CEC 2017 F3, F7, F8; CEC 2022 F1, F4, F5). The functions favoring the 0.4 setting are predominantly heavily multimodal with dense local optima (e.g., F8) or unimodal with high condition numbers (e.g., F7), where an extended exploration budget is critical for escaping deep attraction basins. Conversely, on flat-valley and separable multimodal landscapes, the longer exploitation window provided by the 0.3 setting proves advantageous. Overall, both configurations maintain robust convergence profiles across diverse topologies, empirically validating the 30–40% biological life-cycle proportion that underpins SPO’s parameter design.

#### 4.4.2. Population Size

Appendix A Table A4 reports SPO’s average rank and first-place counts across all 16 functions as a function of population size N∈{20,30,50,100}, with the exploration ratio and iteration limit fixed at 0.4 and T=1000, respectively. The mean runtime per iteration is also reported to quantify computational trade-offs. The average rank improves monotonically as *N* decreases from 100 to 20 (4.00 → 3.94 → 3.62 → 3.12). This rank improvement is driven by the interaction between population size and the stagnation operator hierarchy. With a smaller population, fewer individuals compete for the global best, causing the stagnation thresholds to be reached more quickly. This accelerates the activation of the Carabid, Beauveria, and Parasitoid operators, injecting diversity more aggressively and subsequently improving SPO’s relative standing against the baseline algorithms. Despite the optimal rank achieved at N=20, N=30 is retained as the default setting: the rank difference is modest, and N=30 aligns with standard evaluation protocols in the metaheuristic benchmarking literature, ensuring fair external comparisons. However, for time-constrained applications, N=20 presents a highly efficient alternative.

**Figure A2:**
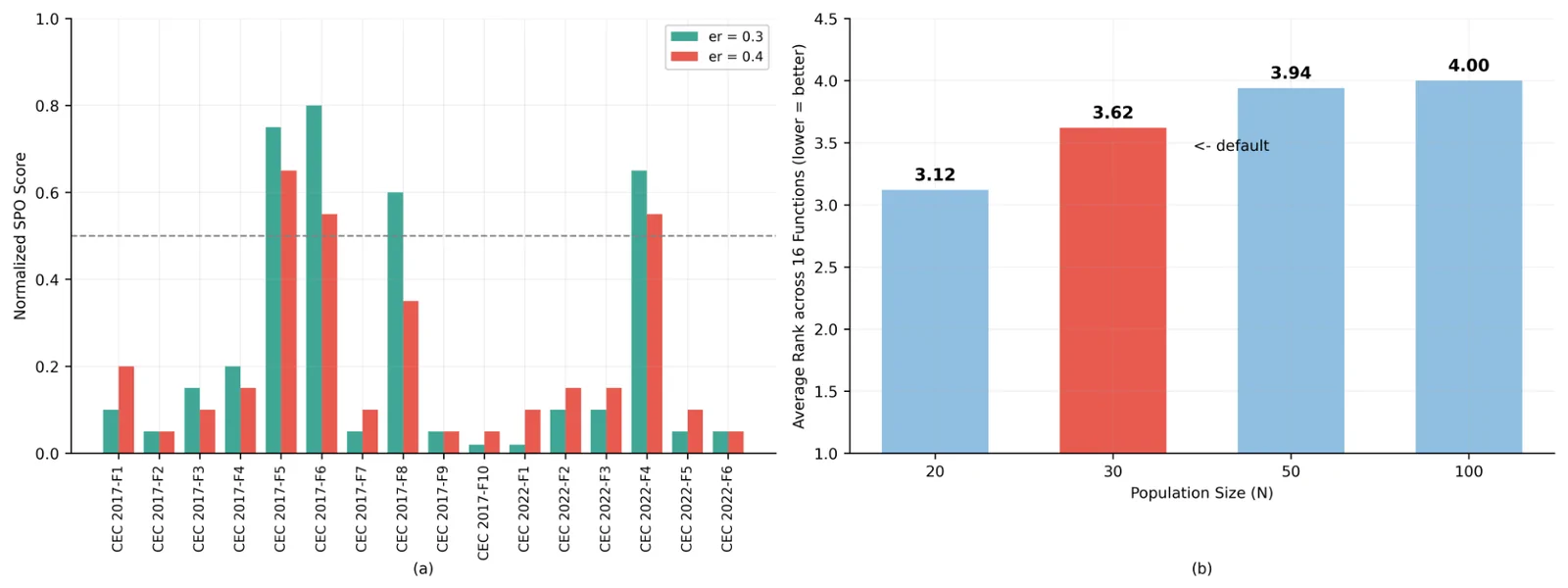
Parameter-sensitivity analysis: (**a**) per-function normalized SPO score for exploration ratios; (**b**) average rank as a function of population size.

Appendix A Figure A2 visually summarizes these sensitivity dynamics. Panel (a) presents the per-function normalized SPO scores under exploration ratios of 0.3 and 0.4, illustrating the topological trade-offs discussed previously: an extended exploration phase (0.4) benefits dense local-optima landscapes, while an extended exploitation phase (0.3) favors flat-valley and separable functions. Panel (b) plots the monotonic rank improvement as population size decreases. The steepest performance gain occurs between N=30 and N=20, demonstrating that SPO’s stagnation-driven recovery mechanism thrives in compact populations where rapid operator escalation can effectively counteract premature convergence.

### 4.5. Extended Comparison with Recent and State-of-the-Art Baselines

The five baselines used in the initial evaluation (GA, PSO, GWO, WOA, DE) serve as well-established classical reference points. To rigorously situate SPO within the contemporary metaheuristic landscape, the comprehensive 16-function benchmark comparison (Table 5) incorporates two additional algorithms: GBFIO, a recently published nature-inspired algorithm sharing SPO’s contemporary context, and L-SHADE, a highly regarded state-of-the-art adaptive differential evolution variant and CEC competition winner. To ensure strict computational parity across differing algorithmic architectures, all evaluations were constrained to an identical objective function evaluation budget (MaxFES = 30,000). For L-SHADE, which natively utilizes an evaluation limit, this budget was applied directly. For GBFIO, the iteration count was scaled down to accurately offset its multiple internal candidate evaluations per generation.

The comprehensive ranking in Table 5 demonstrates L-SHADE’s expected dominance across the suite. As a mature architecture engineered specifically for this class of numerical benchmark, L-SHADE effectively establishes the absolute state-of-the-art performance ceiling. However, measuring SPO against this upper bound highlights its primary comparative achievement: SPO comprehensively outperforms its direct contemporary, GBFIO, and consistently matches or exceeds the classical baselines. By evaluating SPO under these rigorous constraints, the results confirm that its tiered stagnation hierarchy bridges the gap between traditional bio-inspired exploration and the structural robustness required by complex fitness landscapes, establishing a highly competitive position within the current generation of nature-inspired optimizers.

### 4.6. Scalability at Higher Dimensionality (D = 30)

To assess algorithmic scalability as the search space expands, the comprehensive evaluation was repeated at D=30. The experimental protocol remains identical to the D=10 evaluation.

Table 6 reports the D=30 ranking, with the full per-function mean and standard deviation values detailed in Appendix A Table A5 and Table A6. The scaling to D=30 reveals distinct shifts in relative performance dynamics. L-SHADE maintains its absolute dominance (average rank 1.00, 16/16 first-place finishes), demonstrating highly scalable adaptive control. Meanwhile, GA improves markedly from an average rank of 4.25 at D=10 to 2.81 at D=30. This improvement aligns with the recognized scalability of standard crossover-and-mutation mechanisms on higher-dimensional, decomposable landscapes compared to purely swarm-social tracking.

For SPO, the transition to D=30 results in an average rank shift from 3.62 to 4.69. Consequently, its highly competitive D=10 record against GA inverses to a statistical deficit (0 significantly better, 15 significantly worse, 1 tied). This performance drop at higher dimensions directly reflects the theoretical and topological boundaries identified earlier: as *D* increases, the lack of explicit axis-aligned individual memory ($p_{best}$) restricts tracking efficiency on high-dimensional shifted landscapes. Despite these scaling frictions, SPO maintains a decisive statistical advantage over GWO, GBFIO, and PSO, while remaining fully competitive with WOA and DE. This confirms that, although SPO’s primary structural advantage lies in navigating lower-dimensional, highly rugged topologies, its stagnation hierarchy successfully prevents catastrophic failure and outperforms several established baselines even as dimensionality expands.

The scalability profile observed at D=30 points to a specific mechanical limitation rooted in SPO’s parameter design. The stagnation hierarchy is governed by fixed iteration-count thresholds (8, 16, and 24 for the Carabid, Beauveria, and Parasitoid operators, respectively) and a diversity-override threshold computed relative to the initial-population spread (Equation (9)). Crucially, neither metric is defined relative to the problem dimensionality, *D*. As *D* increases from 10 to 30, the expected pairwise distance between randomly sampled individuals grows proportionally with $\sqrt{D}$. Because the stagnation thresholds and the Beauveria operator’s dimensional-repair fraction ($n_{d}=\left\lceil 0.3D\right\rceil$) remain static, the calibration obtained at D=10 (Section 4.4) likely loses efficiency in higher-dimensional spaces. By contrast, GA’s crossover-and-mutation mechanisms lack such dimensionally rigid thresholds, insulating them from this specific scaling friction. Developing dimension-adaptive triggering thresholds for SPO represents a clear avenue for future work to address this geometric bottleneck.

Ultimately, while SPO’s relative performance against GA diminishes at higher dimensions, this shift isolates a specific topological vulnerability rather than a generalized algorithmic breakdown. SPO’s statistical advantage over contemporary and classical baselines—including GWO, GBFIO, and PSO—is strictly maintained or even amplified at D=30. This confirms that, although its static parameterization currently limits its tracking efficiency against purely evolutionary crossover operators in high-dimensional domains, SPO’s stagnation hierarchy remains a fundamentally robust search mechanism across expanding search spaces.

### 4.7. Discussion

The aggregate results establish a clear topological boundary for SPO. It performs best on multimodal and composite functions with irregular fitness landscapes (e.g., F4, F5, and the broader CEC 2022 suite), where the tiered stagnation hierarchy provides a graduated diversity-injection mechanism. Conversely, it struggles on narrow-valley unimodal functions (F6, F8, F9) and ill-conditioned rotated functions, where an explicit individual memory term ($p_{best}$) is highly advantageous. Furthermore, DE’s superiority on separable, near-convex functions (F1, F2, F6, F9 of CEC 2017) indicates that, while SPO’s operator hierarchy effectively navigates deceptive multimodality, it is mechanically extraneous on simple, well-behaved landscapes where classical differential mutation can converge directly.

The Lévy flight exploration phase is the primary driver of SPO’s strong performance on F4 and the closely related F5. The heavy-tailed displacement distribution enables the algorithm to traverse large fitness plateaus and escape local optima that typically trap PSO and GA during early iterations. Once the population localizes around a promising region, the stagnation hierarchy activates; specifically, the Beauveria operator’s targeted dimensional repair facilitates fine-grained exploitation without the severe diversity loss associated with full random restarts.

PSO’s superiority on F8, F9, and C22-F5 highlights a specific geometric limitation. These functions share a dense anisotropic local-optima structure where the global optimum is strongly aligned with individual coordinate axes. PSO’s $p_{best}$ term tracks the historical best of each particle in every dimension independently, yielding a distinct axis-aligned tracking advantage. In contrast, SPO’s differential swarm term encodes population-relative directional information that is inherently direction-agnostic, rendering it less effective on axis-aligned topologies. Integrating a covariance-matrix adaptation or a dimension-wise local search mechanism presents a logical future direction to bridge this gap.

A notable exception to SPO’s generally compact rank profile is CEC 2017-F7, where it ranks fourth among the classical baselines (WOA > PSO > GWO > SPO > DE > GA). F7 is a Schwefel-type function with a deceptive global structure, locating the true optimum geometrically distant from the centroid of good solutions. On this specific landscape, SPO’s population-relative differential term and stagnation-triggered re-initializations prove less effective than WOA’s spiral-encircling mechanism, empirically defining the boundaries of SPO’s centroid-oriented search logic on highly non-centroid-aligned topologies.

Mechanistically, the sensitivity analysis validates SPO’s foundational design principles. The exploration ratio results confirm that the biologically motivated default (0.4) optimally balances search dynamics across a diverse benchmark set, with the [0.3, 0.4] interval providing robust generalization. Furthermore, the population size analysis demonstrates that the stagnation operator hierarchy confers inherent resilience to small population environments. This is a significant structural advantage over algorithms relying solely on social interaction (e.g., PSO), where N<30 typically precipitates rapid premature convergence.

Finally, the statistical divergence between raw mean-fitness comparisons and Bonferroni-corrected significance warrants attention. While GA achieves a numerically lower mean than SPO on 6 of the 16 functions, none of these differences achieve statistical significance following the Bonferroni correction ($\alpha^{\prime }=0.01$). This gap stems from the high coefficient-of-variation (CV) observed for GA across multiple CEC 2017 functions (Appendix A Table A1 and Table A2), indicating severe per-run fitness dispersion. The nonparametric Wilcoxon rank-sum test is inherently conservative under such high within-group variance, confirming that GA’s apparent numerical advantage on those specific functions is highly unstable, whereas SPO maintains statistically reliable convergence across independent runs.

### 4.8. Real-World Application: Sunn Pest Monitoring-Network Placement

Sunn pest (*Eurygaster integriceps* Puton) is among the most economically damaging pests of winter wheat across the Middle East, Central Asia, and the Balkans, with reported yield and quality losses of up to 50% in untreated fields [41]. Early and spatially accurate detection of the pest’s spring re-entry into cereal fields is central to integrated pest management (IPM) programs, because it allows targeted, threshold-based insecticide application rather than blanket spraying across an entire production zone. In practice, detection relies on a budget-constrained network of field-scouting/monitoring stations that cannot cover an entire cultivated area exhaustively and must therefore be positioned to capture the areas of highest expected infestation risk first. This section evaluates SPO, alongside the seven competitor algorithms benchmarked earlier, on the monitoring-network placement problem, thereby offering a domain-native validation that directly connects the algorithm’s performance to its biological motivation.

#### 4.8.1. Problem Formulation

A network of N monitoring stations must be placed within a square production zone of side length L to maximize the risk-weighted fraction of the cultivated area brought within detection range, subject to a minimum inter-station spacing constraint reflecting scouting-logistics limits. As in the CEC and engineering-design experiments above, a candidate layout is encoded as a flat, continuous decision vector shown in Equation (19):

(19) $$X=\left[x_{1},y_{1},x_{2},y_{2},\ldots ,x_{N},y_{N}\right]\in {\left[0,L\right]}^{2N}$$

Sunn pest overwinters in adjacent highland and shrub habitat and undergoes a well-documented mass vertical migration back into wheat fields each spring (Section 3.1.1; [40]). Infestation risk is therefore not spatially uniform: sub-parcels nearest the field edge adjacent to overwintering habitat face the earliest and heaviest pressure, decaying with distance into the field interior. This is encoded as an exponential risk-weight gradient over a discretized grid of M sub-parcel centroids p_k, anchored at the migration-entry edge (y = 0) (Equation (20)):   

(20) $$w\left(p_{k}\right)=w_{min}+\left(1-w_{min}\right)\cdot exp\left(\left(-y_{k}/\lambda \right)\right)$$

where y_k is the sub-parcel’s distance from the migration-entry edge, λ is a decay length scale, and w_min is a residual background risk retained far from the edge. This weighting is an explicit, stated modelling assumption motivated by the documented migration behaviour, not a calibrated dispersal model, and is reported as such rather than presented as a validated field measurement.

A sub-parcel is considered covered if it lies within radius r of at least one station. No calibrated attraction-radius figure specific to *E. integriceps* monitoring traps was identified in the literature search accompanying this study; rather than assert an unsupported figure, r is treated as a stated, transparent modelling choice (corresponding to an approximately 15 ha circular catchment per station, broadly consistent with the order of magnitude of regional cereal-pest scouting-density conventions) rather than a measured constant. The risk-weighted coverage fraction is defined as (Equation (21)):

(21) $$C\left(X\right)=\frac{\sum_{k}w\left(p_{k}\right)\cdot 1\left[\exists i:∥X_{i}-p_{k}∥\leq r\right]}{\sum_{k}w\left(p_{k}\right)}$$

and the objective (minimized) combines uncovered weighted risk with a penalty for violating the minimum inter-station spacing dmin and the field boundary (Equation (22)):

(22) $$f\left(X\right)=\left(1-C\left(X\right)\right)+\lambda_{1}\sum_{i<j}{\left(\frac{max\left(\left(0,d_{min}-∥X_{i}-X_{j}∥\right)\right)}{d_{min}}\right)}^{2}+\lambda_{2}\sum_{i}{\left(boundary\;violation\right)}^{2}$$

With N = 25 stations, a 3000 m × 3000 m production zone (~900 ha), and r = 220 m, even a perfect non-overlapping hexagonal packing covers at most ≈42% of the zone—full coverage is infeasible under this scouting budget by construction. The optimization problem is therefore genuinely one of prioritization: which ~42% of the field, weighted by migration-risk, should be covered first.

#### 4.8.2. Experimental Setup

The demand surface is discretized on a 30 × 30 grid (900 sub-parcels). The risk-weight gradient uses λ=1500 m and $w_{\min}=0.15$. A minimum inter-station spacing of $d_{\min}=250$ m is enforced identically across all eight algorithms via the exterior penalty in Equation (22), consistent with the constraint-handling approach used throughout this study. All eight algorithms were evaluated using a population size of $N_{\mathrm{pop}}=30$, with 10 independent runs each and no problem-specific hyperparameter retuning. To ensure strict computational parity across differing architectures, the total function evaluation budget was uniformly equalized across all eight algorithms (equivalent to T=200 iterations for the standard baselines), exactly as detailed in Section 4.5.

#### 4.8.3. Results

Table 7 reports, for each algorithm, the constraint-feasibility rate, the mean and best risk-weighted coverage achieved, and the resulting rank.

The spatial layout problem imposes a comparatively loose minimum spacing constraint relative to the search space area (300 pairwise constraints among 25 stations across a 900 ha zone). Consequently, the exterior penalty method (Equation (22)) effectively enforces feasibility for seven of the eight algorithms, including SPO, which satisfy the spacing constraint in 100% of independent runs. The sole exception is GBFIO, which exhibits a feasibility rate of only 5/10. Because constraint satisfaction is near-universal across the established baselines, the primary differentiator on this task is optimization quality (risk-weighted coverage) rather than feasibility. This confirms that SPO’s core search mechanics seamlessly translate to penalized applied domains without inducing the severe constraint-violation failures observed in its contemporary counterpart, GBFIO.

**Figure 2:**
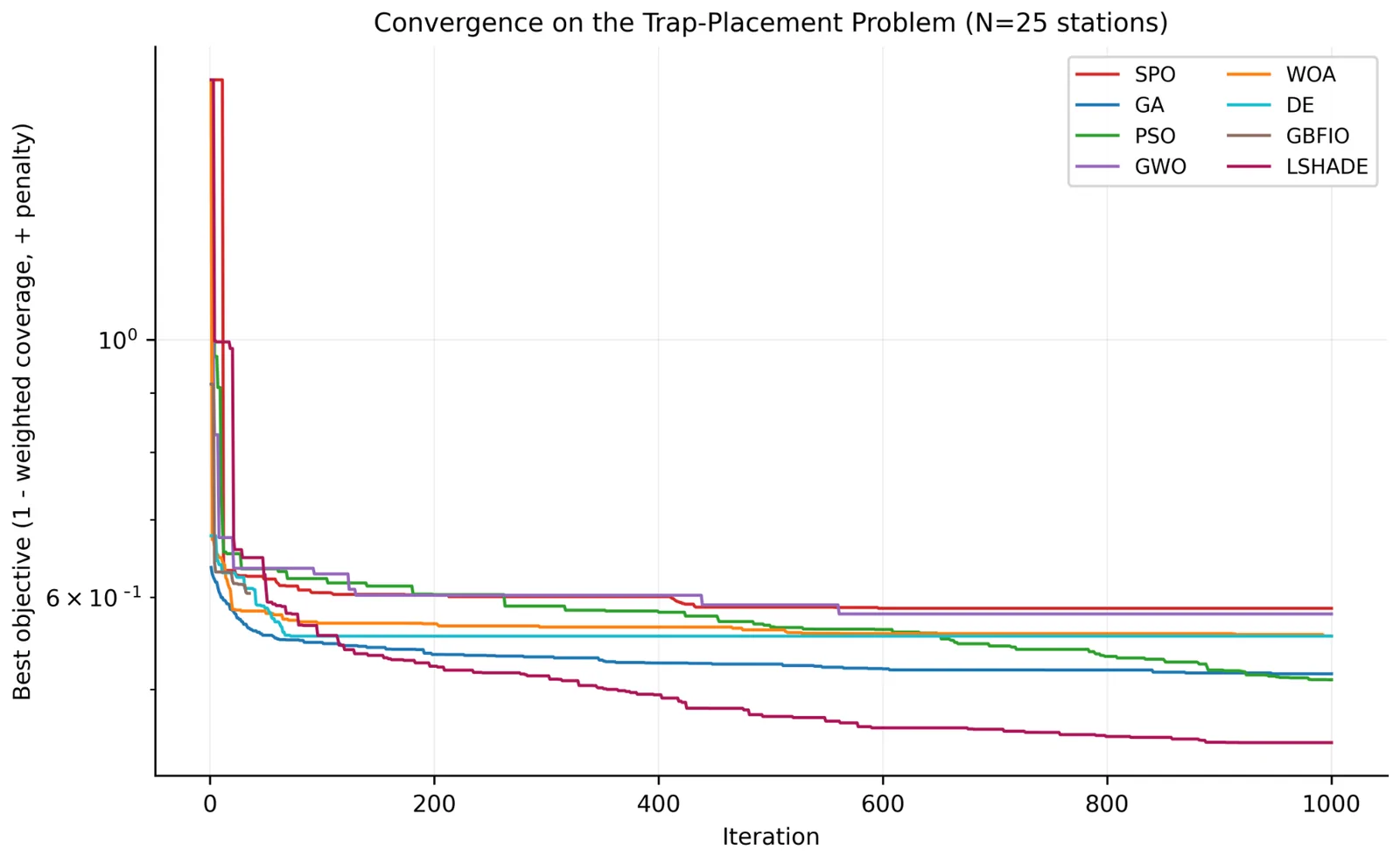
Convergence of SPO vs. GA, PSO, GWO, WOA, DE on the trap-placement problem (N = 25 stations), log-scale best objective, one representative run per algorithm.

**Figure 3:**
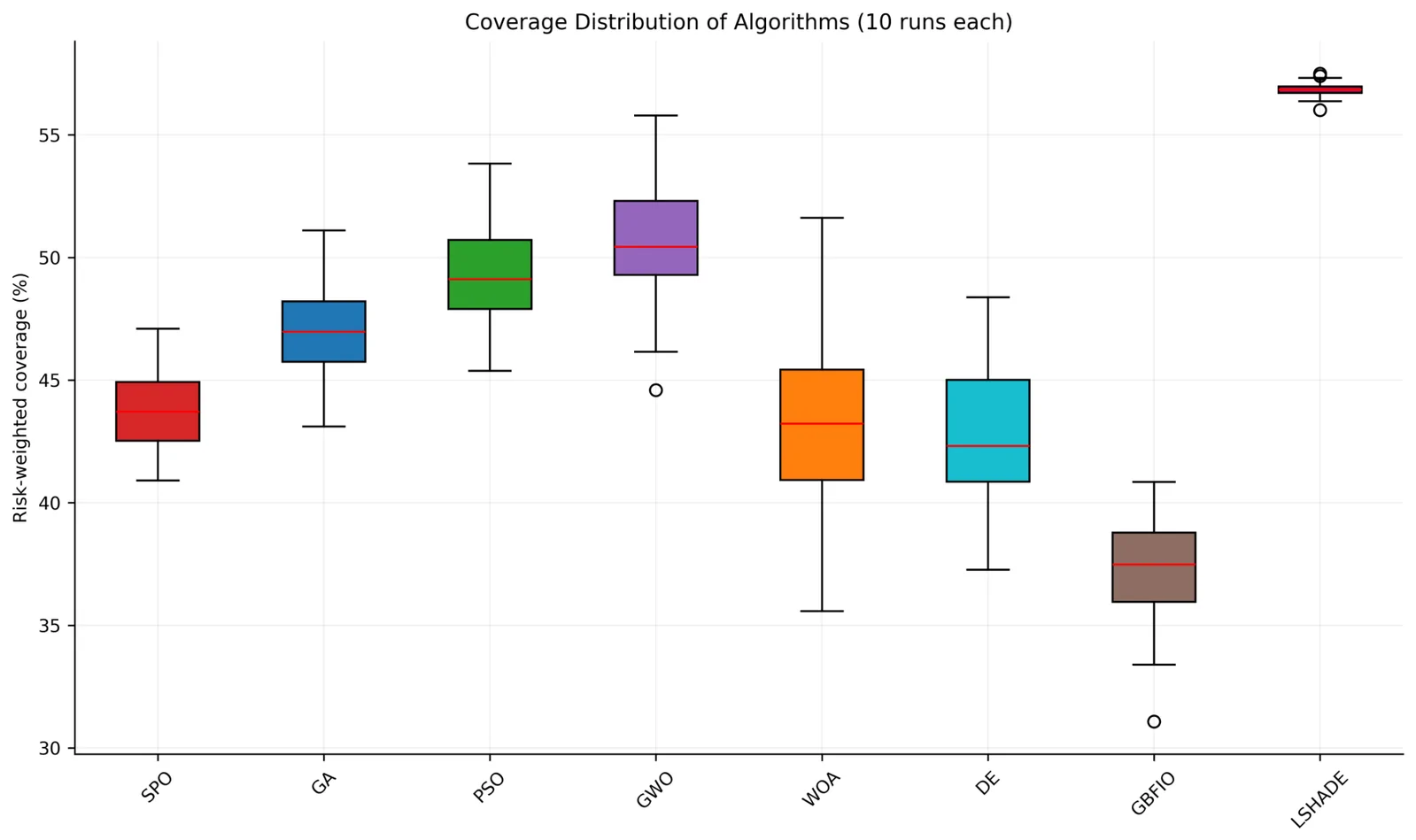
Risk-weighted coverage (%) distribution by algorithm, 10 independent runs each.

**Figure 4:**
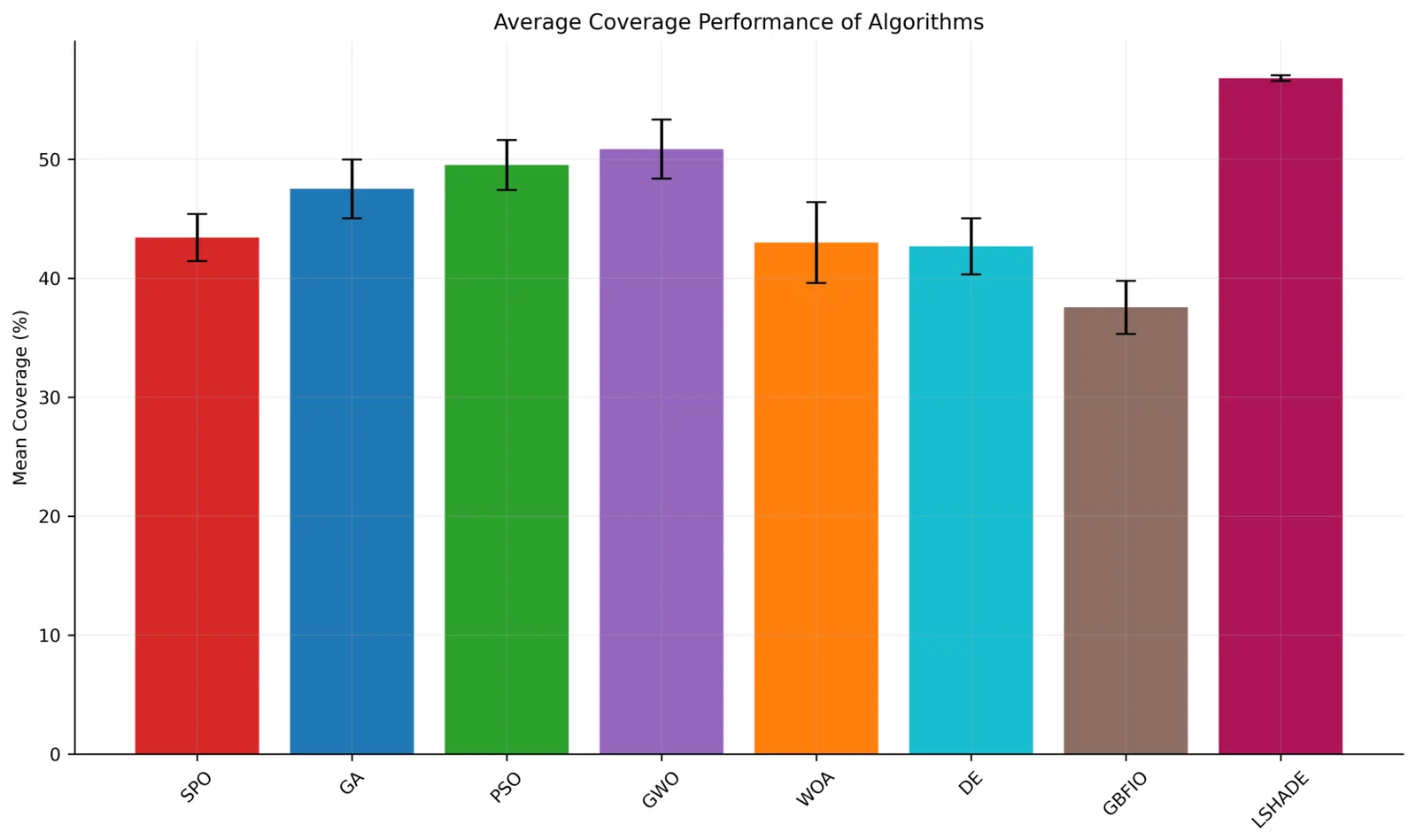
Mean risk-weighted coverage (%) achieved by each algorithm, ranked.

**Figure 5:**
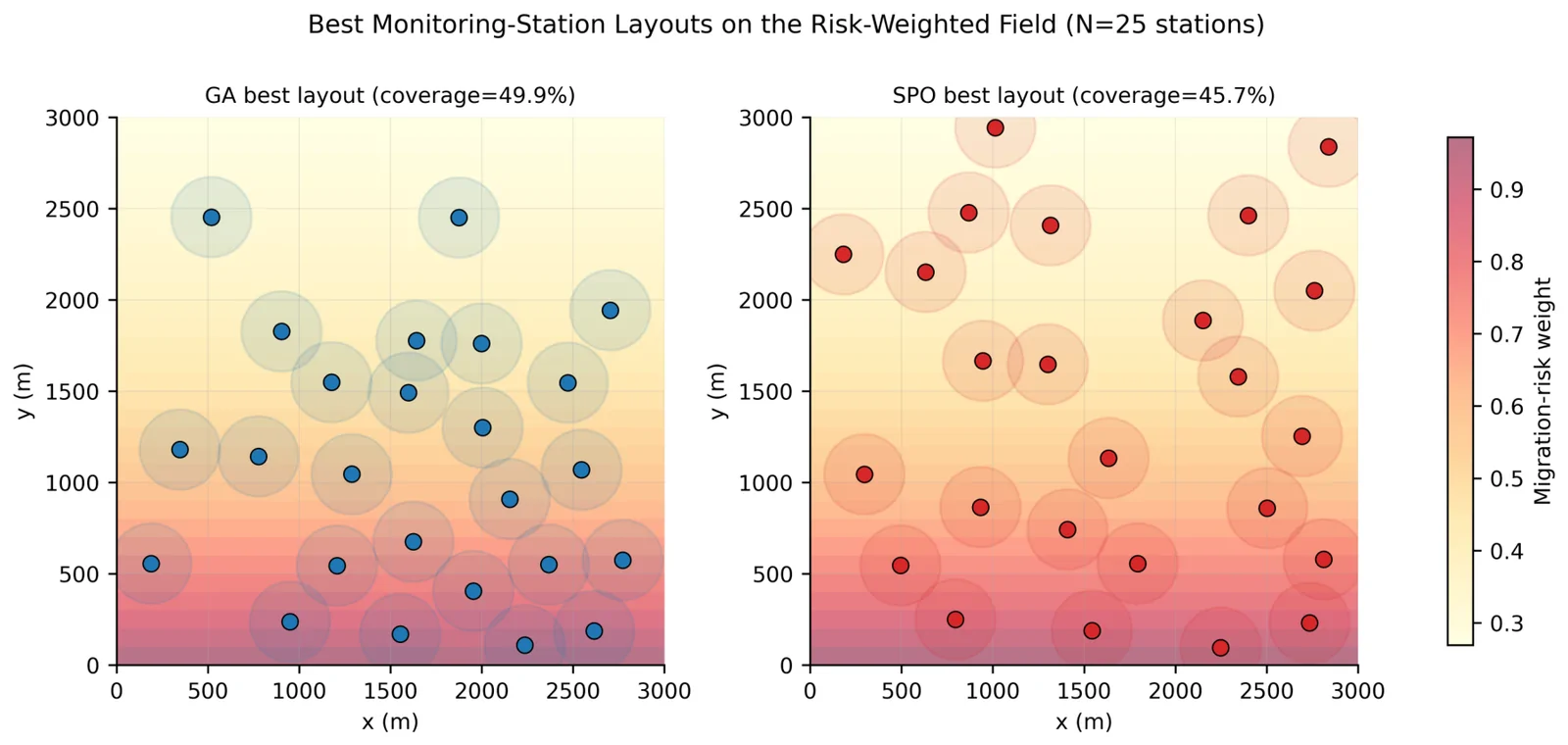
Best monitoring-station layouts found by GA and SPO, superimposed on the migration-risk weight surface (darker = higher risk, concentrated near the migration-entry edge at y = 0); circles show each station’s 220 m catchment.

Figure 2, Figure 3 and Figure 4 present the convergence curves, coverage distributions, and mean risk-weighted coverage for each algorithm, respectively, while Figure 5 visualizes the optimal layouts found by SPO and GA. L-SHADE achieves the highest mean coverage (56.8% ± 0.2%), followed by GWO (50.9% ± 2.5%), PSO (49.5% ± 2.1%), and GA (47.5% ± 2.5%). SPO ranks fifth overall (43.4% ± 2.0%), outperforming WOA, DE, and GBFIO (37.6% ± 2.2%). A Bonferroni-corrected Mann–Whitney U test ($\alpha^{\prime }=0.05/7\approx 0.0071$) confirms that SPO’s coverage is significantly lower than L-SHADE, GWO, and PSO, and significantly higher than GBFIO. The numerical differences against GA, WOA, and DE do not achieve statistical significance, primarily due to the substantial run-to-run variance exhibited by those classical baselines. Ultimately, while SPO does not dominate this specific spatial deployment task against the specialized tracking mechanisms of PSO or L-SHADE, it maintains a statistically robust, middle-tier performance, consistently generating highly feasible optimization layouts across independent runs.

As illustrated in Figure 5, the optimal layouts generated by both GA and SPO successfully concentrate the majority of monitoring stations within the high-risk region near the migration-entry edge, while distributing a minority of stations toward the field interior to capture residual background risk. Both algorithms thus recover the qualitatively correct spatial prioritization strategy. The primary distinction between the two lies in local packing efficiency along the high-risk boundary. GA achieves slightly more uniform spacing among edge-proximal stations, effectively minimizing redundant catchment overlap. Conversely, SPO’s layout exhibits marginally higher coverage overlap in these dense regions. This micro-structural difference indicates that, while SPO excels at identifying the macro-level optimal zones, its population-relative differential mechanism is slightly less efficient at fine-tuning localized spatial packing compared to the precise micro-adjustments afforded by classical mutation operators.

#### 4.8.4. Real-World Application Discussion

The application of SPO to the spatial monitoring problem yields distinct insights into its operational capabilities. In terms of absolute coverage quality, SPO ranks fifth out of the eight evaluated algorithms. While it is outperformed by the highly specialized tracking mechanisms of L-SHADE, GWO, and PSO, it remains statistically competitive with GA, WOA, and DE, and significantly outperforms its contemporary counterpart, GBFIO. More importantly, SPO’s constraint-handling mechanism proves exceptionally robust: it achieved a 100% feasibility rate across all independent runs, successfully navigating the penalized search space to satisfy the minimum-spacing constraints without the severe violation rates observed in GBFIO (5/10). This confirms that, while SPO’s population-relative differential mechanism may trail explicit $p_{best}$ trackers in raw coverage maximization, it reliably translates its core search stability to constrained, real-world topologies.

From an applied IPM perspective, the qualitative layouts generated by SPO align perfectly with established Sunn pest bioecology and regional survey practices [40,53]. The optimization framework successfully prioritizes high-risk, overwintering-adjacent field edges before distributing the remaining stations toward the interior. For practitioners, this spatial prioritization has direct operational consequences. By concentrating a limited 25-station scouting budget where migration-driven infestations first emerge, growers can transition from blanket precautionary spraying to threshold-based, targeted insecticide applications at specific sub-parcels.

While the choice of optimizer dictates the strict numerical efficiency of this coverage, the layouts produced by SPO validate the underlying risk-weighted framework, demonstrating the algorithm’s capacity to generate both mathematically feasible and ecologically sound monitoring networks. For future operational deployment, transitioning these SPO-derived layouts to the field requires only the calibration of the theoretical coverage-radius and risk-decay parameters against local trapping-efficacy trials. Ultimately, this application confirms SPO’s viability as a robust decision-support tool for precision agriculture, effectively bridging the gap between theoretical metaheuristic design and practical resource allocation.

### 4.9. Limitations

The current formulation of SPO presents specific mechanical and topological boundaries that define its operational scope:

- Dimensional scalability: SPO’s parameterization relies on fixed iteration-count thresholds and static dimensional-repair fractions. While highly effective at D=10, this rigid architecture introduces scaling friction at higher dimensions (D=30). This is exacerbated by the O(DlogD) computational cost inherent in the Beauveria operator’s sorting mechanism, which scales less gracefully than purely evolutionary operators.
- Topological boundaries: the absence of an explicit individual memory term ($p_{best}$) limits tracking efficiency on dense, axis-aligned, or ill-conditioned landscapes. Furthermore, the stagnation hierarchy introduces overhead that proves mechanically extraneous on perfectly separable functions, where classical differential mutation can converge directly.
- Parameter generalization: the baseline triggering thresholds and exploration ratios were calibrated alongside the primary evaluation suite. Validating these static parameters across entirely disjoint or structurally distinct test suites is required to confirm broad out-of-sample generalization.
- Applied simulation parameters: in the spatial trap-placement application, while SPO successfully navigated the geometric constraints, the mathematical definitions of risk-decay and minimum catchment spacing remain theoretically illustrative rather than empirically field-calibrated.

### 4.10. Future Work

Based on these structural boundaries, primary directions for future research include:

- Dimension-adaptive architecture: formulating the stagnation thresholds and dimensional-repair fractions as dynamic functions of the problem dimensionality (*D*) to maintain diversity-injection efficiency across expanding search spaces.
- Covariance and positional memory: integrating explicit individual positional tracking or covariance-matrix adaptation to enhance exploitation specifically on anisotropic, axis-aligned, and rotated topologies.
- Component ablation and cross-validation: conducting a systematic, operator-level ablation study to isolate the precise statistical contribution of the Carabid, Beauveria, and Parasitoid phases, coupled with parameter tuning on a disjoint benchmark suite (e.g., CEC 2025) to ensure rigorous cross-domain generalization.
- Empirical IPM calibration: grounding the theoretical spatial monitoring framework by calibrating the optimization parameters against local Sunn pest trapping-efficacy trials and geographic data prior to operational agricultural deployment.

## 5. Conclusions

This study introduced the Sunn Pest Optimization (SPO) algorithm, a novel metaheuristic grounded in the behavioral ecology of *Eurygaster integriceps*. By abstracting seasonal migration, foraging, natural enemy pressure, and overwintering survival into strictly defined algorithmic mechanisms—namely, Lévy flight exploration, differential swarm exploitation, a tiered stagnation hierarchy, and elitism—SPO structurally couples spatial population density to dynamic operator selection.

Evaluated across 16 CEC benchmark functions at D=10, SPO established a highly competitive standing. It statistically outperformed both contemporary and classical baselines, including GBFIO, GWO, and WOA, while maintaining robust convergence on complex multimodal and composite landscapes. Although it naturally trails the absolute state-of-the-art upper bound defined by L-SHADE, SPO’s performance maps clear topological boundaries, demonstrating specific mechanical limitations only on axis-aligned or separable geometries where explicit individual positional memory ($p_{best}$) provides a distinct advantage.

At higher dimensionalities (D=30), SPO maintains a strict statistical advantage over several baselines and contemporary algorithms, though its static parameter thresholds introduce expected scaling friction relative to pure crossover mechanisms. Crucially, in the applied monitoring-network deployment problem, SPO demonstrated exceptional constraint-handling reliability—achieving a 100% feasibility rate—and successfully generated ecologically valid, risk-prioritized layouts that align with established agronomic practices.

Ultimately, SPO demonstrates that ecologically motivated heuristics, when directly mapped to mathematically transparent operations, yield highly capable and structurally robust optimization engines. Future development will focus on integrating dimension-adaptive thresholds and covariance-matrix-aware tracking to systematically expand SPO’s efficiency across high-dimensional and explicitly rotated topological spaces.

## Figures and Tables

**Table 1:** Observed biological features of *Eurygaster integriceps*, functional rationale for their inclusion in SPO, and their algorithmic counterparts.

Observed Biological Feature	Functional Rationale for Inclusion	SPO Counterpart
Seasonal vertical migration exhibiting heavy-tailed, long-range dispersal (most individuals travel short distances; a few travel very long distances)	An exploration operator capable of simultaneously producing local and global jumps across the search space is required	Lévy flight exploration (Section 3.4.1)
Migration and settlement occupy only ~30–40% of the total life cycle	The exploration phase must be shorter than exploitation; excessive exploration prevents convergence within the budget	explore_ratio = 0.4 (Section 3.4)
Intra-field group feeding: individuals respond to both the best patch and the relative positions of conspecifics	Sole dependence on Xbest underperforms on rotated functions; differential population information compensates for missing gradient direction	Differential swarm exploitation (Section 3.4.2)
Beauveria bassiana infection is partial (organ-level) and potentially reversible	Targeted repair of only the most deviant dimensions, combined with conditional acceptance, minimizes information loss compared to full random restart	Beauveria operator (Section 3.5.4)
Predation by Carabidae disproportionately eliminates poorly positioned individuals; the predated individual is replaced by a fully new one	Targeted, unconditional re-initialization of low-fitness individuals prevents premature convergence	Carabid operator (Section 3.5.3)
Trissolcus Parasitoid pressure is collective, sudden, and density-dependent	A large-scale, rapid diversity injection is required; it must trigger both periodically and when spatial density exceeds a threshold	Parasitoid operator + diversity-override (Section 3.5.5)
Only a small but high-quality cohort survives to the next season	Preserving the best configurations is necessary, but the ratio must remain low to avoid over-reducing diversity	Elitism, elite_ratio = 0.1 (Section 3.6)

**Table 2:** Biological–algorithmic analogy table for SPO.

Biological Behaviour	SPO Component	Mathematical Counterpart
Spring migration (overwintering site → field)	Exploration phase	Equation (3)
Intra-field foraging and competition	Exploitation phase	Equation (6)
Carabidae predator pressure	Carabid operator	Equations (10) and (11)
Beauveria bassiana fungal infection	Beauveria operator	Equations (12)–(14)
Trissolcus Parasitoid egg destruction	Parasitoid operator	Equations (15)–(17)
Spatial crowding (field saturation)	Diversity trigger	Equations (7)–(9)
Robust adults returning to overwintering sites	Elitism	Equation (18)

**Table 3:** Fitness evaluation cost structure for all compared algorithms.

Algorithm	Initialization	Per-Iteration (Fixed)	Dynamic Operator Cost	Total FE Formula
GA	N	N	—	N + (T·N)
PSO	N	N	—	N + (T·N)
GWO	N	N	—	N + (T·N)
WOA	N	N	—	N + (T·N)
DE	N	N	—	N + (T·N)
SPO	N	N	K(N·0.20) + A(N) + P(N·0.30)	N + (T·N) + Σ(K,A,P)
GBFIO	N	28 N	—	N + (T_*G*_·28N) ≈ N + (T·N)
L-SHADE	N	N_*P*_(*g*)	—	FE_*max*_ = N + (T·N)

**Table 4:** SPO algorithm parameters, default values, and equation references.

Parameter	Default	Equation & Biological Counterpart
N	30	Population size—number of Sunn pest individuals
T	1000	Maximum iterations—season duration
$\alpha_{1,\max}$	1.5	Equation (6) maximum social coefficient
$\alpha_{2}$	0.5	Equation (6) differential swarm coefficient
$w_{\max}/w_{\min}$	0.9/0.2	Equation (2) wind coefficient linear decay bounds
β	1.5	Equations (4) and (5) Lévy distribution tail index (1 < β ≤ 2)
s	0.01	Equation (3) Lévy step scale factor
explore_ratio	0.4	Exploration/exploitation phase transition ratio (40% of T)
ε	$10^{-4}$	Equation (9) spatial diversity threshold ratio
carabid_ratio	0.2	Equation (10) fraction of individuals eliminated
carabid_stagnation	8	Equation (10) stagnation counter threshold
beauveria_dim_ratio	0.3	Equation (12) fraction of dimensions repaired
beauveria_stagnation	16	Equation (12) stagnation counter threshold
Parasitoid_kill_ratio	0.3	Equation (15) fraction of individuals re-initialized
Parasitoid_stagnation	24	Equation (15) stagnation counter threshold and diversity-override target
elite_ratio	0.1	Equation (18) overwintering memory (elitism) ratio

**Table 5:** Overall eight-algorithm ranking and two-sided Wilcoxon rank-sum test results (N = 30, explore_ratio = 0.4, T = 1000, 30 runs, 16 CEC 2017/CEC 2022 functions).

Algorithm	Avg. Rank	Overall	Sig. Better	Sig. Worse	Tie
LSHADE	1.00	1st	0	16	0
SPO	3.62	2nd	—	—	—
GA	4.25	3rd	6	5	5
WOA	4.88	4th	10	4	2
PSO	4.94	5th	3	7	6
DE	5.25	6th	7	8	1
GBFIO	6.00	7th	12	1	3
GWO	6.06	8th	8	0	8

**Table 6:** Extended eight-algorithm ranking at D = 30 (N = 30, explore_ratio = 0.4, T = 1000, 30 runs, 16 CEC 2017/CEC 2022 functions). Sig. Better/Worse/Tie defined as in Table 5.

Algorithm	Avg. Rank	Overall	Sig. Better	Sig. Worse	Tie
LSHADE	1.00	1st	0	16	0
GA	2.81	2nd	0	15	1
DE	4.38	3rd	3	8	5
WOA	4.56	4th	8	5	3
SPO	4.69	5th	—	—	—
PSO	5.25	6th	7	2	7
GWO	6.00	7th	11	2	3
GBFIO	7.31	8th	13	0	3

**Table 7:** Coverage and feasibility summary on the trap-placement problem, extended to eight algorithms (N = 25 stations, 10 runs each).

Algorithm	Feasible (n/10)	Mean Coverage (%) ± SD	Best Coverage (%)	Rank
LSHADE	10/10	56.83 ± 0.24	57.33	1
GWO	10/10	50.86 ± 2.48	52.99	2
PSO	10/10	49.53 ± 2.10	52.07	3
GA	10/10	47.52 ± 2.47	51.00	4
SPO	10/10	43.42 ± 1.99	46.72	5
WOA	10/10	43.00 ± 3.41	47.29	6
DE	10/10	42.68 ± 2.36	46.46	7
GBFIO	5/10	37.55 ± 2.23	41.06	8

## Data Availability

The original code and data presented in the study are openly available in GitHub at: https://github.com/sametdiri/sunnpestoptimization (accessed on 10 September 2026).

## Appendix A. Complete Benchmark Results and Parameter-Sensitivity Analyses

This appendix provides the complete per-function benchmark results and parameter-sensitivity analyses summarized in Section 4.2, Section 4.3 and Section 4.4. Table A1 and Table A2 report the full mean ± standard deviation results for the comprehensive eight-algorithm evaluation, with the corresponding overall ranking detailed in Table 5 (Section 4.1). Figure A1 provides the corresponding fitness-distribution details for all eight evaluated algorithms. Table A3 and Figure A2a report SPO’s internal sensitivity to the exploration-ratio setting (0.3 vs. 0.4), while Table A4 and Figure A2 report SPO’s average rank against the full eight-algorithm field as a function of population size, summarized in Section 4.4. Table A5 and Table A6 report the full mean ± standard deviation results underlying the D = 30 scalability comparison of Section 4.6 (Table 6), in the same format as Table A1 and Table A2.

**Table A1:** Mean ± standard deviation over 30 independent runs on CEC 2017 (N=30, explore_ratio = 0.4, T=1000), extended to all eight compared algorithms. Bold indicates the best mean per function. For F7, lower (more negative) is better.

Fn.	SPO	GA	PSO	GWO	WOA	DE	GBFIO	LSHADE
F1	$4.93\times 10^{-3}\pm 5.43\times 10^{-3}$	$5.79\times 10^{-7}\pm 8.49\times 10^{-7}$	$3.93\times 10^{+1}\pm 1.50\times 10^{+2}$	$2.34\times 10^{+1}\pm 7.14\times 10^{+1}$	$1.20\times 10^{-3}\pm 1.61\times 10^{-3}$	$2.63\times 10^{-24}\pm 1.07\times 10^{-23}$	$4.93\times 10^{-1}\pm 5.81\times 10^{-1}$	0
F2	$2.83\times 10^{+5}\pm 2.80\times 10^{+5}$	$6.07\times 10^{+5}\pm 5.31\times 10^{+5}$	$5.31\times 10^{+5}\pm 4.76\times 10^{+5}$	$8.41\times 10^{+5}\pm 1.03\times 10^{+6}$	$1.71\times 10^{+6}\pm 1.43\times 10^{+6}$	$9.06\times 10^{+4}\pm 1.56\times 10^{+5}$	$3.04\times 10^{+6}\pm 2.45\times 10^{+6}$	$8.11\times 10^{+2}\pm 2.24\times 10^{+3}$
F3	$4.34\times 10^{+1}\pm 1.25\times 10^{+1}$	$3.34\times 10^{+1}\pm 1.31\times 10^{+1}$	$1.25\times 10^{+2}\pm 3.99\times 10^{+2}$	$6.14\times 10^{+1}\pm 4.40\times 10^{+1}$	$6.03\times 10^{+1}\pm 1.95\times 10^{+1}$	$2.36\times 10^{+2}\pm 4.86\times 10^{+2}$	$6.07\times 10^{+1}\pm 1.06\times 10^{+1}$	$6.34\times 10^{+0}\pm 2.26\times 10^{+0}$
F4	$1.90\times 10^{+1}\pm 4.65\times 10^{+0}$	$2.00\times 10^{+1}\pm 1.29\times 10^{-3}$	$1.97\times 10^{+1}\pm 3.72\times 10^{+0}$	$1.98\times 10^{+1}\pm 3.70\times 10^{+0}$	$2.01\times 10^{+1}\pm 1.23\times 10^{-1}$	$2.01\times 10^{+1}\pm 2.72\times 10^{+0}$	$2.05\times 10^{+1}\pm 9.33\times 10^{-2}$	$1.89\times 10^{+1}\pm 4.47\times 10^{+0}$
F5	$5.18\times 10^{+2}\pm 2.15\times 10^{+3}$	$4.60\times 10^{+2}\pm 1.48\times 10^{+3}$	$6.19\times 10^{+5}\pm 1.89\times 10^{+6}$	$1.48\times 10^{+5}\pm 4.01\times 10^{+5}$	$6.21\times 10^{+4}\pm 9.64\times 10^{+4}$	$5.35\times 10^{+4}\pm 2.58\times 10^{+5}$	$7.90\times 10^{+4}\pm 1.41\times 10^{+5}$	$4.09\times 10^{+0}\pm 1.95\times 10^{+1}$
F6	$6.52\times 10^{-1}\pm 2.79\times 10^{-1}$	$6.50\times 10^{-1}\pm 2.88\times 10^{-1}$	$2.65\times 10^{-1}\pm 1.41\times 10^{-1}$	$6.38\times 10^{-1}\pm 1.65\times 10^{-1}$	$3.12\times 10^{-1}\pm 1.92\times 10^{-1}$	$2.36\times 10^{-1}\pm 1.97\times 10^{-1}$	$7.43\times 10^{-1}\pm 1.35\times 10^{-1}$	$7.43\times 10^{-3}\pm 7.09\times 10^{-3}$
F7	$-3.20\times 10^{+3}\pm 6.16\times 10^{+2}$	$-2.43\times 10^{+3}\pm 7.04\times 10^{+2}$	$-3.39\times 10^{+3}\pm 7.74\times 10^{+2}$	$-3.27\times 10^{+3}\pm 6.59\times 10^{+2}$	$-4.04\times 10^{+3}\pm 5.79\times 10^{+2}$	$-2.94\times 10^{+3}\pm 9.06\times 10^{+2}$	$-3.75\times 10^{+3}\pm 7.65\times 10^{+2}$	$-4.22\times 10^{+3}\pm 5.20\times 10^{+2}$
F8	$9.54\times 10^{+0}\pm 1.23\times 10^{+1}$	$1.22\times 10^{+3}\pm 4.42\times 10^{+2}$	$4.42\times 10^{+0}\pm 2.25\times 10^{+1}$	$2.16\times 10^{+1}\pm 4.61\times 10^{+1}$	$2.56\times 10^{+2}\pm 3.78\times 10^{+2}$	$4.10\times 10^{+2}\pm 3.41\times 10^{+2}$	$1.46\times 10^{+1}\pm 1.51\times 10^{+1}$	$6.04\times 10^{-2}\pm 1.48\times 10^{-1}$
F9	$6.57\times 10^{+1}\pm 3.08\times 10^{+2}$	$7.32\times 10^{+1}\pm 2.23\times 10^{+2}$	$5.97\times 10^{-7}\pm 1.13\times 10^{-6}$	$1.80\times 10^{+3}\pm 2.09\times 10^{+3}$	$5.02\times 10^{+1}\pm 8.00\times 10^{+1}$	$4.90\times 10^{-24}\pm 2.28\times 10^{-23}$	$5.68\times 10^{+3}\pm 3.59\times 10^{+3}$	$1.35\times 10^{-29}\pm 4.47\times 10^{-29}$
F10	$2.922\times 10^{+2}\pm 9.805\times 10^{-3}$	$2.922\times 10^{+2}\pm 1.223\times 10^{-2}$	$2.923\times 10^{+2}\pm 1.481\times 10^{-2}$	$2.923\times 10^{+2}\pm 1.536\times 10^{-2}$	$2.922\times 10^{+2}\pm 9.757\times 10^{-3}$	$2.922\times 10^{+2}\pm 2.359\times 10^{-2}$	$2.923\times 10^{+2}\pm 2.064\times 10^{-2}$	$2.922\times 10^{+2}\pm 5.863\times 10^{-3}$

**Table A2:** Mean ± standard deviation over 30 independent runs on CEC 2022 (N=30, explore_ratio = 0.4, T=1000), extended to all eight compared algorithms. Bold indicates the best mean per function.

Fn.	SPO	GA	PSO	GWO	WOA	DE	GBFIO	LSHADE
F1	$1.87\times 10^{+0}\pm 6.03\times 10^{+0}$	$2.65\times 10^{+0}\pm 3.33\times 10^{+0}$	$6.02\times 10^{+1}\pm 3.29\times 10^{+2}$	$1.87\times 10^{+3}\pm 2.43\times 10^{+3}$	$3.54\times 10^{+2}\pm 3.60\times 10^{+2}$	$2.38\times 10^{+2}\pm 1.01\times 10^{+3}$	$5.73\times 10^{+3}\pm 2.60\times 10^{+3}$	$9.87\times 10^{-30}\pm 5.34\times 10^{-29}$
F2	$8.01\times 10^{+3}\pm 3.42\times 10^{+4}$	$1.05\times 10^{+2}\pm 4.55\times 10^{+2}$	$1.63\times 10^{+5}\pm 6.19\times 10^{+5}$	$4.13\times 10^{+5}\pm 4.35\times 10^{+5}$	$1.65\times 10^{+5}\pm 3.39\times 10^{+5}$	$8.14\times 10^{+4}\pm 4.46\times 10^{+5}$	$2.18\times 10^{+5}\pm 2.12\times 10^{+5}$	$1.06\times 10^{+0}\pm 1.79\times 10^{+0}$
F3	$2.48\times 10^{+2}\pm 4.43\times 10^{+2}$	$1.17\times 10^{+2}\pm 2.16\times 10^{+2}$	$1.16\times 10^{+4}\pm 1.89\times 10^{+4}$	$3.25\times 10^{+5}\pm 1.55\times 10^{+6}$	$3.55\times 10^{+3}\pm 3.87\times 10^{+3}$	$3.23\times 10^{+5}\pm 1.76\times 10^{+6}$	$4.57\times 10^{+3}\pm 5.32\times 10^{+3}$	$6.22\times 10^{-1}\pm 1.69\times 10^{-1}$
F4	$4.44\times 10^{+1}\pm 1.65\times 10^{+1}$	$3.87\times 10^{+1}\pm 1.84\times 10^{+1}$	$1.16\times 10^{+2}\pm 2.29\times 10^{+2}$	$8.69\times 10^{+1}\pm 1.06\times 10^{+2}$	$7.06\times 10^{+1}\pm 2.97\times 10^{+1}$	$1.93\times 10^{+2}\pm 1.93\times 10^{+2}$	$5.92\times 10^{+1}\pm 1.08\times 10^{+1}$	$5.87\times 10^{+0}\pm 1.84\times 10^{+0}$
F5	$2.19\times 10^{+1}\pm 2.50\times 10^{+1}$	$1.39\times 10^{+3}\pm 5.45\times 10^{+2}$	$6.75\times 10^{+0}\pm 2.17\times 10^{+1}$	$6.31\times 10^{+1}\pm 1.42\times 10^{+2}$	$3.16\times 10^{+2}\pm 3.14\times 10^{+2}$	$3.23\times 10^{+2}\pm 2.10\times 10^{+2}$	$1.68\times 10^{+1}\pm 1.34\times 10^{+1}$	$5.97\times 10^{-3}\pm 2.27\times 10^{-2}$
F6	$1.037\times 10^{+2}\pm 1.041\times 10^{+0}$	$1.028\times 10^{+2}\pm 1.037\times 10^{+0}$	$1.063\times 10^{+2}\pm 9.581\times 10^{+0}$	$1.039\times 10^{+2}\pm 5.060\times 10^{+0}$	$1.049\times 10^{+2}\pm 2.048\times 10^{+0}$	$1.258\times 10^{+2}\pm 3.552\times 10^{+1}$	$1.046\times 10^{+2}\pm 5.881\times 10^{-1}$	$1.007\times 10^{+2}\pm 2.440\times 10^{-1}$

**Table A3:** Sensitivity of SPO mean fitness to explore_ratio (N=30, T=1000, and 30 runs). Bold indicates the lower (better) mean.

Fn.	*er* = 0.3 (Mean ± Std)	*er* = 0.4 (Mean ± Std)	Better	Suite
F1	$2.23\times 10^{-3}\pm 2.37\times 10^{-3}$	$4.93\times 10^{-3}\pm 5.43\times 10^{-3}$	er=0.3	CEC 2017
F2	$2.68\times 10^{+5}\pm 3.70\times 10^{+5}$	$2.83\times 10^{+5}\pm 2.80\times 10^{+5}$	er=0.3	CEC 2017
F3	$4.84\times 10^{+1}\pm 1.66\times 10^{+1}$	$4.34\times 10^{+1}\pm 1.25\times 10^{+1}$	er=0.4	CEC 2017
F4	$1.84\times 10^{+1}\pm 5.94\times 10^{+0}$	$1.90\times 10^{+1}\pm 4.65\times 10^{+0}$	er=0.3	CEC 2017
F5	$6.99\times 10^{+1}\pm 1.61\times 10^{+2}$	$5.18\times 10^{+2}\pm 2.15\times 10^{+3}$	er=0.3	CEC 2017
F6	$5.54\times 10^{-1}\pm 2.79\times 10^{-1}$	$6.52\times 10^{-1}\pm 2.79\times 10^{-1}$	er=0.3	CEC 2017
F7	$-2.98\times 10^{+3}\pm 9.35\times 10^{+2}$	$-3.20\times 10^{+3}\pm 6.16\times 10^{+2}$	er=0.4	CEC 2017
F8	$2.35\times 10^{+1}\pm 5.29\times 10^{+1}$	$9.54\times 10^{+0}\pm 1.23\times 10^{+1}$	er=0.4	CEC 2017
F9	$3.27\times 10^{+0}\pm 1.17\times 10^{+1}$	$6.57\times 10^{+1}\pm 3.08\times 10^{+2}$	er=0.3	CEC 2017
F10	$2.922\times 10^{+2}\pm 9.759\times 10^{-3}$	$2.922\times 10^{+2}\pm 9.805\times 10^{-3}$	er=0.3	CEC 2017
F1	$1.12\times 10^{+1}\pm 5.96\times 10^{+1}$	$1.87\times 10^{+0}\pm 6.03\times 10^{+0}$	er=0.4	CEC 2022
F2	$2.37\times 10^{+3}\pm 1.26\times 10^{+4}$	$8.01\times 10^{+3}\pm 3.42\times 10^{+4}$	er=0.3	CEC 2022
F3	$2.03\times 10^{+2}\pm 4.35\times 10^{+2}$	$2.48\times 10^{+2}\pm 4.43\times 10^{+2}$	er=0.3	CEC 2022
F4	$4.49\times 10^{+1}\pm 1.64\times 10^{+1}$	$4.44\times 10^{+1}\pm 1.65\times 10^{+1}$	er=0.4	CEC 2022
F5	$4.12\times 10^{+1}\pm 6.61\times 10^{+1}$	$2.19\times 10^{+1}\pm 2.50\times 10^{+1}$	er=0.4	CEC 2022
F6	$1.034\times 10^{+2}\pm 1.158\times 10^{+0}$	$1.037\times 10^{+2}\pm 1.041\times 10^{+0}$	er=0.3	CEC 2022

**Table A4:** Effect of population size on SPO’s average rank against the full eight-algorithm field (exploration ratio = 0.4, T=1000, and 30 runs).

*N* (Population Size)	Average SPO Rank	First Place	Runtime/Iteration (ms)
20	3.12	1/16	0.77
30	3.62	0/16	1.09
50	3.94	0/16	1.72
100	4.00	1/16	3.37

**Table A5:** Mean ± standard deviation over 30 independent runs on CEC 2017 at D=30 (N=30, explore_ratio = 0.4, T=1000), for all eight compared algorithms. Bold indicates the best mean per function. For F7, lower (more negative) is better.

Fn.	SPO	GA	PSO	GWO	WOA	DE	GBFIO	LSHADE
F1	$5.04\times 10^{+1}\pm 9.25\times 10^{+1}$	$2.92\times 10^{+0}\pm 6.60\times 10^{-1}$	$2.93\times 10^{+3}\pm 2.32\times 10^{+3}$	$1.33\times 10^{+3}\pm 1.23\times 10^{+3}$	$1.47\times 10^{+1}\pm 1.01\times 10^{+1}$	$3.49\times 10^{-3}\pm 1.91\times 10^{-2}$	$2.07\times 10^{+3}\pm 8.93\times 10^{+2}$	$4.21\times 10^{-21}\pm 1.44\times 10^{-20}$
F2	$1.70\times 10^{+7}\pm 1.12\times 10^{+7}$	$7.67\times 10^{+6}\pm 4.07\times 10^{+6}$	$1.59\times 10^{+7}\pm 1.12\times 10^{+7}$	$2.18\times 10^{+7}\pm 1.94\times 10^{+7}$	$2.50\times 10^{+7}\pm 1.04\times 10^{+7}$	$4.05\times 10^{+6}\pm 3.06\times 10^{+6}$	$7.48\times 10^{+7}\pm 2.43\times 10^{+7}$	$5.62\times 10^{+5}\pm 3.80\times 10^{+5}$
F3	$3.90\times 10^{+2}\pm 8.48\times 10^{+1}$	$2.66\times 10^{+2}\pm 4.32\times 10^{+1}$	$3.41\times 10^{+3}\pm 2.54\times 10^{+3}$	$1.93\times 10^{+3}\pm 1.45\times 10^{+3}$	$6.76\times 10^{+2}\pm 1.95\times 10^{+2}$	$3.62\times 10^{+3}\pm 1.28\times 10^{+3}$	$2.32\times 10^{+3}\pm 7.14\times 10^{+2}$	$7.16\times 10^{+1}\pm 2.95\times 10^{+1}$
F4	$2.10\times 10^{+1}\pm 9.26\times 10^{-2}$	$2.05\times 10^{+1}\pm 1.14\times 10^{-1}$	$2.10\times 10^{+1}\pm 7.27\times 10^{-2}$	$2.10\times 10^{+1}\pm 5.10\times 10^{-2}$	$2.03\times 10^{+1}\pm 1.79\times 10^{-1}$	$2.10\times 10^{+1}\pm 8.66\times 10^{-2}$	$2.10\times 10^{+1}\pm 6.30\times 10^{-2}$	$2.03\times 10^{+1}\pm 2.29\times 10^{-1}$
F5	$4.04\times 10^{+5}\pm 5.94\times 10^{+5}$	$2.01\times 10^{+4}\pm 3.43\times 10^{+4}$	$1.45\times 10^{+7}\pm 7.47\times 10^{+7}$	$2.04\times 10^{+7}\pm 2.09\times 10^{+7}$	$3.45\times 10^{+5}\pm 5.29\times 10^{+5}$	$3.48\times 10^{+5}\pm 1.14\times 10^{+6}$	$4.84\times 10^{+7}\pm 2.85\times 10^{+7}$	$3.89\times 10^{+2}\pm 1.63\times 10^{+3}$
F6	$7.98\times 10^{-1}\pm 3.19\times 10^{-1}$	$2.66\times 10^{-1}\pm 7.31\times 10^{-2}$	$2.06\times 10^{+0}\pm 6.05\times 10^{-1}$	$1.43\times 10^{+0}\pm 4.19\times 10^{-1}$	$4.21\times 10^{-1}\pm 2.06\times 10^{-1}$	$1.15\times 10^{-1}\pm 4.60\times 10^{-1}$	$1.48\times 10^{+0}\pm 2.10\times 10^{-1}$	$5.09\times 10^{-3}\pm 7.23\times 10^{-3}$
F7	$-5.21\times 10^{+3}\pm 2.03\times 10^{+3}$	$-6.36\times 10^{+3}\pm 1.89\times 10^{+3}$	$-6.78\times 10^{+3}\pm 2.13\times 10^{+3}$	$-4.60\times 10^{+3}\pm 3.73\times 10^{+3}$	$-8.48\times 10^{+3}\pm 2.07\times 10^{+3}$	$-8.62\times 10^{+3}\pm 2.10\times 10^{+3}$	$-9.81\times 10^{+2}\pm 1.30\times 10^{+3}$	$-9.24\times 10^{+3}\pm 1.10\times 10^{+3}$
F8	$4.13\times 10^{+3}\pm 1.11\times 10^{+3}$	$9.99\times 10^{+2}\pm 8.34\times 10^{+2}$	$5.82\times 10^{+2}\pm 5.78\times 10^{+2}$	$1.88\times 10^{+3}\pm 1.02\times 10^{+3}$	$6.98\times 10^{+3}\pm 1.95\times 10^{+3}$	$3.91\times 10^{+3}\pm 1.47\times 10^{+3}$	$4.39\times 10^{+3}\pm 1.70\times 10^{+3}$	$7.85\times 10^{+1}\pm 6.40\times 10^{+1}$
F9	$1.55\times 10^{+4}\pm 6.39\times 10^{+3}$	$4.47\times 10^{+3}\pm 2.65\times 10^{+3}$	$1.57\times 10^{+4}\pm 7.05\times 10^{+3}$	$2.95\times 10^{+4}\pm 6.89\times 10^{+3}$	$2.35\times 10^{+4}\pm 5.67\times 10^{+3}$	$2.34\times 10^{+3}\pm 8.38\times 10^{+3}$	$6.31\times 10^{+4}\pm 1.07\times 10^{+4}$	$2.31\times 10^{+2}\pm 3.90\times 10^{+2}$
F10	$5.34\times 10^{+2}\pm 7.68\times 10^{-2}$	$5.34\times 10^{+2}\pm 3.80\times 10^{-2}$	$5.34\times 10^{+2}\pm 2.72\times 10^{-2}$	$5.34\times 10^{+2}\pm 2.93\times 10^{-2}$	$5.34\times 10^{+2}\pm 2.49\times 10^{-2}$	$5.34\times 10^{+2}\pm 7.93\times 10^{-2}$	$5.34\times 10^{+2}\pm 2.92\times 10^{-2}$	$5.34\times 10^{+2}\pm 1.32\times 10^{-2}$

**Table A6:** Mean ± standard deviation over 30 independent runs on CEC 2022 at D=30 (N=30, explore_ratio = 0.4, T=1000), for all eight compared algorithms. Bold indicates the best mean per function.

Fn.	SPO	GA	PSO	GWO	WOA	DE	GBFIO	LSHADE
F1	$1.82\times 10^{+4}\pm 7.20\times 10^{+3}$	$5.49\times 10^{+3}\pm 3.11\times 10^{+3}$	$1.73\times 10^{+4}\pm 1.07\times 10^{+4}$	$3.28\times 10^{+4}\pm 9.70\times 10^{+3}$	$3.47\times 10^{+4}\pm 7.16\times 10^{+3}$	$4.95\times 10^{+3}\pm 1.28\times 10^{+4}$	$7.07\times 10^{+4}\pm 1.30\times 10^{+4}$	$3.51\times 10^{+2}\pm 9.01\times 10^{+2}$
F2	$4.20\times 10^{+5}\pm 5.06\times 10^{+5}$	$2.45\times 10^{+4}\pm 3.24\times 10^{+4}$	$1.14\times 10^{+7}\pm 4.05\times 10^{+7}$	$3.51\times 10^{+7}\pm 5.02\times 10^{+7}$	$3.57\times 10^{+5}\pm 2.43\times 10^{+5}$	$3.76\times 10^{+6}\pm 1.94\times 10^{+7}$	$5.52\times 10^{+7}\pm 8.23\times 10^{+7}$	$5.70\times 10^{+1}\pm 1.08\times 10^{+2}$
F3	$1.24\times 10^{+6}\pm 2.01\times 10^{+6}$	$6.71\times 10^{+3}\pm 5.42\times 10^{+3}$	$2.10\times 10^{+7}\pm 4.51\times 10^{+7}$	$2.55\times 10^{+7}\pm 3.92\times 10^{+7}$	$1.27\times 10^{+5}\pm 3.91\times 10^{+5}$	$2.40\times 10^{+7}\pm 1.30\times 10^{+8}$	$4.64\times 10^{+7}\pm 7.41\times 10^{+7}$	$2.64\times 10^{+2}\pm 7.16\times 10^{+2}$
F4	$3.71\times 10^{+2}\pm 7.14\times 10^{+1}$	$2.49\times 10^{+2}\pm 4.66\times 10^{+1}$	$2.02\times 10^{+3}\pm 2.58\times 10^{+3}$	$1.45\times 10^{+3}\pm 1.24\times 10^{+3}$	$7.21\times 10^{+2}\pm 1.78\times 10^{+2}$	$3.35\times 10^{+3}\pm 1.32\times 10^{+3}$	$2.15\times 10^{+3}\pm 6.54\times 10^{+2}$	$7.53\times 10^{+1}\pm 3.06\times 10^{+1}$
F5	$5.20\times 10^{+3}\pm 1.54\times 10^{+3}$	$1.61\times 10^{+3}\pm 9.36\times 10^{+2}$	$7.60\times 10^{+2}\pm 6.22\times 10^{+2}$	$2.09\times 10^{+3}\pm 1.37\times 10^{+3}$	$6.72\times 10^{+3}\pm 2.70\times 10^{+3}$	$4.64\times 10^{+3}\pm 1.96\times 10^{+3}$	$4.00\times 10^{+3}\pm 1.86\times 10^{+3}$	$7.26\times 10^{+1}\pm 7.79\times 10^{+1}$
F6	$1.25\times 10^{+2}\pm 5.43\times 10^{+0}$	$1.18\times 10^{+2}\pm 3.23\times 10^{+0}$	$2.84\times 10^{+2}\pm 2.02\times 10^{+2}$	$2.29\times 10^{+2}\pm 8.68\times 10^{+1}$	$1.51\times 10^{+2}\pm 1.62\times 10^{+1}$	$3.58\times 10^{+2}\pm 9.40\times 10^{+1}$	$2.60\times 10^{+2}\pm 6.92\times 10^{+1}$	$1.05\times 10^{+2}\pm 1.53\times 10^{+0}$

## References

[1] Mirjalili S. (2018). Evolutionary Algorithms and Neural Networks: Theory and Applications.

[2] Holland J.H. (1975). Adaptation in Natural and Artificial Systems.

[3] Kennedy J., Eberhart R. (1995). Particle swarm optimization. Proceedings of ICNN’95—International Conference on Neural Networks.

[4] Storn R., Price K. (1997). Differential evolution—A simple and efficient heuristic for global optimization over continuous spaces. J. Glob. Optim..

[5] Mirjalili S., Mirjalili S.M., Lewis A. (2014). Grey wolf optimizer. Adv. Eng. Softw..

[6] Mirjalili S., Lewis A. (2016). The whale optimization algorithm. Adv. Eng. Softw..

[7] Yang X.-S., Deb S. (2009). Cuckoo search via Lévy flights. 2009 World Congress on Nature & Biologically Inspired Computing.

[8] Wolpert D.H., Macready W.G. (1997). No free lunch theorems for optimization. IEEE Trans. Evol. Comput..

[9] Karaboga D. (2005). An Idea Based on Honey Bee Swarm for Numerical Optimization.

[10] Rajwar K., Deep K., Das S. (2023). An exhaustive review of the metaheuristic algorithms for search and optimization: Taxonomy, applications, and open challenges. Artif. Intell. Rev..

[11] Dokeroglu T., Canturk D., Kucukyilmaz T. (2025). A survey on pioneering metaheuristic algorithms between 2019 and 2024 (Version 1) [Preprint]. arXiv.

[12] Mirjalili S. (2016). SCA: A sine cosine algorithm for solving optimization problems. Knowl.-Based Syst..

[13] Mirjalili S., Gandomi A.H., Mirjalili S.Z., Saremi S., Faris H., Mirjalili S.M. (2017). Salp swarm algorithm: A bio-inspired optimizer for engineering design problems. Adv. Eng. Softw..

[14] Rao R.V., Savsani V.J., Vakharia D.P. (2011). Teaching–learning-based optimization: A novel method for constrained mechanical design optimization problems. Comput.-Aided Des..

[15] Abdollahzadeh B., Gharehchopogh F.S., Mirjalili S. (2021). African vultures optimization algorithm: A new nature-inspired metaheuristic algorithm for global optimization problems. Comput. Ind. Eng..

[16] Dehghani M., Aly M., Rodriguez J., Sheybani E., Javidi G. (2025). A novel nature-inspired optimization algorithm: Grizzly bear fat increase optimizer. Biomimetics.

[17] Kang J., Ma Z. (2025). Red-crowned crane optimization: A novel biomimetic metaheuristic algorithm for engineering applications. Biomimetics.

[18] Chen C., Cao L., Chen B., Chen Y., Wu X. (2025). A dual-mechanism enhanced secretary bird optimization algorithm and its application in engineering optimization. Biomimetics.

[19] Cao Y., Lv H., Cui Y., Wu Z., Zhang Q. (2026). Improved superb fairy-wren optimization algorithm and its application. Biomimetics.

[20] Varna F.T., Husbands P. (2024). Two new bio-inspired particle swarm optimisation algorithms for single-objective continuous variable problems based on eavesdropping and altruistic animal behaviours. Biomimetics.

[21] Yıldız B.S., Kumar S., Pholdee N., Bureerat S., Sait S.M., Yildiz A.R. (2022). A new chaotic Lévy flight distribution optimization algorithm for solving constrained engineering problems. Expert Syst..

[22] Yang X.S. (2010). Engineering Optimization: An Introduction with Metaheuristic Applications.

[23] Yang X.S., Deb S. (2010). Engineering optimisation by cuckoo search. Int. J. Math. Model. Numer. Optim..

[24] Yang X.S. (2020). Nature-Inspired Optimization Algorithms.

[25] Lee C.-Y., Yao X. (2004). Evolutionary programming using mutations based on the Lévy probability distribution. IEEE Trans. Evol. Comput..

[26] Yang X.-S. (2012). Flower pollination algorithm for global optimization. Unconventional Computation and Natural Computation.

[27] Reynolds A.M., Frye M.A. (2007). Free-flight odor tracking in *Drosophila* is consistent with an optimal intermittent scale-free search. PLoS ONE.

[28] Zhong C., Li G., Meng Z. (2022). Beluga whale optimization: A novel nature-inspired metaheuristic algorithm. Knowl.-Based Syst..

[29] Haklı H., Uğuz H. (2014). A novel particle swarm optimization algorithm with Levy flight. Appl. Soft Comput..

[30] Salimi H. (2015). Stochastic fractal search: A powerful metaheuristic algorithm. Knowl.-Based Syst..

[31] Liang J.J., Qin A.K., Suganthan P.N., Baskar S. (2006). Comprehensive learning particle swarm optimizer for global optimization of multimodal functions. IEEE Trans. Evol. Comput..

[32] Zhang J., Sanderson A.C. (2009). JADE: Adaptive differential evolution with optional external archive. IEEE Trans. Evol. Comput..

[33] Church R., ReVelle C. (1974). The maximal covering location problem. Pap. Reg. Sci. Assoc..

[34] Ferentinos K.P., Tsiligiridis T.A. (2007). Adaptive design optimization of wireless sensor networks using genetic algorithms. Comput. Netw..

[35] Jawad H., Nordin R., Gharghan S., Jawad A., Ismail M. (2017). Energy-efficient wireless sensor networks for precision agriculture: A review. Sensors.

[36] Wu L., Qu J., Shi H., Li P. (2022). Node deployment optimization for wireless sensor networks based on virtual force-directed particle swarm optimization algorithm and evidence theory. Entropy.

[37] Shoaib M., Sadeghi-Niaraki A., Ali F., Hussain I., Khalid S. (2025). Leveraging deep learning for plant disease and pest detection: A comprehensive review and future directions. Front. Plant Sci..

[38] Faiçal B.S., Freitas H., Gomes P.H., Mano L.Y., Pessin G., de Carvalho A.C.P.L.F., Krishnamachari B., Ueyama J. (2017). An adaptive approach for UAV-based pesticide spraying in dynamic environments. Comput. Electron. Agric..

[39] Alonso Campos J.C., Jiménez-Bello M.A., Martínez Alzamora F. (2020). Real-time energy optimization of irrigation scheduling by parallel multi-objective genetic algorithms. Agric. Water Manag..

[40] Lodos N. (1961). Türkiye, Irak, İran ve Suriye’de Süne (Eurygaster integriceps Put.) Problemi Üzerinde Incelemeler.

[41] Davari A., Parker B.L. (2018). A review of research on sunn pest *Eurygaster integriceps* Puton (Hemiptera: Scutelleridae) management published 2004–2016. J.-Asia-Pac. Entomol..

[42] Critchley B.R. (1998). Literature review of sunn pest *Eurygaster integriceps* Put. (Hemiptera, Scutelleridae). Crop Prot..

[43] Conti E., Colazza S. (2012). Chemical ecology of egg parasitoids associated with true bugs. Psyche A J. Entomol..

[44] Babaroğlu N.E., Akci E., Çulcu M., Yalçın F. (2020). Süne ve Mücadelesi.

[45] Shi Y., Eberhart R.C. (1998). Parameter selection in particle swarm optimization. Evolutionary Programming VII.

[46] Mantegna R.N. (1994). Fast, accurate algorithm for numerical simulation of Lévy stable stochastic processes. Phys. Rev. E.

[47] Tanabe R., Fukunaga A.S. (2014). Improving the search performance of SHADE using linear population size reduction. 2014 IEEE Congress on Evolutionary Computation (CEC).

[48] Yang Z., Tang K., Yao X. (2008). Large scale evolutionary optimization using cooperative coevolution. Inf. Sci..

[49] Chen W., Weise T., Yang Z., Tang K. (2010). Large-scale global optimization using cooperative coevolution with variable interaction learning. Parallel Problem Solving from Nature, PPSN XI.

[50] Omidvar M.N., Li X., Mei Y., Yao X. (2014). Cooperative co-evolution with differential grouping for large scale optimization. IEEE Trans. Evol. Comput..

[51] Mengshoel O.J., Flogard E.L., Yu T., Riege J. (2022). Understanding the cost of fitness evaluation for subset selection. Proceedings of the Genetic and Evolutionary Computation Conference.

[52] Sari Z., Yildirim M. (2026). A complexity calculation method for large scale optimization with evolutionary algorithms and metaheuristics. Concurr. Comput. Pract. Exp..

[53] Duman M., Alaserhat İ. (2025). Assessment of *Eurygaster integriceps* parameters to improve integrated pest management in Southeastern Türkiye wheat fields. Sci. Rep..

